# Integrating mean and variance heterogeneities to identify differentially expressed genes

**DOI:** 10.1186/s12859-016-1393-y

**Published:** 2016-12-06

**Authors:** Weiwei Ouyang, Qiang An, Jinying Zhao, Huaizhen Qin

**Affiliations:** 1 Department of Global Biostatistics and Data Science, Tulane University School of Public Health and Tropical Medicine, 1440 Canal Street, Suite 2001, New Orleans, LA 70112 USA; 2 Department of Epidemiology, Tulane University School of Public Health and Tropical Medicine, 1440 Canal Street, New Orleans, LA 70112 USA; 3 Department of Epidemiology, College of Public Health and Health Professions and College of Medicine, University of Florida, 2004 Mowry Rd, Gainesville, FL 32610 USA

**Keywords:** Functional genomics studies, MVDE genes, Integrative heterogeneity test, Latent confounders, Latent biomarkers

## Abstract

**Background:**

In functional genomics studies, tests on mean heterogeneity have been widely employed to identify differentially expressed genes with distinct mean expression levels under different experimental conditions. Variance heterogeneity (aka, the difference between condition-specific variances) of gene expression levels is simply neglected or calibrated for as an impediment. The mean heterogeneity in the expression level of a gene reflects one aspect of its distribution alteration; and variance heterogeneity induced by condition change may reflect another aspect. Change in condition may alter both mean and some higher-order characteristics of the distributions of expression levels of susceptible genes.

**Results:**

In this report, we put forth a conception of mean-variance differentially expressed (MVDE) genes, whose expression means and variances are sensitive to the change in experimental condition. We mathematically proved the null independence of existent mean heterogeneity tests and variance heterogeneity tests. Based on the independence, we proposed an integrative mean-variance test (IMVT) to combine gene-wise mean heterogeneity and variance heterogeneity induced by condition change. The IMVT outperformed its competitors under comprehensive simulations of normality and Laplace settings. For moderate samples, the IMVT well controlled type I error rates, and so did existent mean heterogeneity test (i.e., the Welch t test (WT), the moderated Welch t test (MWT)) and the procedure of separate tests on mean and variance heterogeneities (SMVT), but the likelihood ratio test (LRT) severely inflated type I error rates. In presence of variance heterogeneity, the IMVT appeared noticeably more powerful than all the valid mean heterogeneity tests. Application to the gene profiles of peripheral circulating B raised solid evidence of informative variance heterogeneity. After adjusting for background data structure, the IMVT replicated previous discoveries and identified novel experiment-wide significant MVDE genes.

**Conclusions:**

Our results indicate tremendous potential gain of integrating informative variance heterogeneity after adjusting for global confounders and background data structure. The proposed informative integration test better summarizes the impacts of condition change on expression distributions of susceptible genes than do the existent competitors. Therefore, particular attention should be paid to explicitly exploit the variance heterogeneity induced by condition change in functional genomics analysis.

**Electronic supplementary material:**

The online version of this article (doi:10.1186/s12859-016-1393-y) contains supplementary material, which is available to authorized users.

## Background

Typically, comparative microarray experiments analyze expressions of thousands to tens of thousands of genes. A core challenge is to identify statistically significant genes of biologically meaningful changes in expression levels under different conditions. Differentially expressed genes may help identify disease biomarkers that are important for the diagnosis of multiple diseases [1, 2]. There are several existent mean heterogeneity tests for identifying differentially expressed genes. The Student *t* test (ST) has been widely applied as a standard routine for identifying mean differentially expressed (MDE) genes in two-condition experiments [3]. The null hypothesis of this test is mean homogeneity *H*_01_: the testing gene has identical mean expression level under the two conditions. It assumes variance homogeneity *H*_02_: the testing gene has identical variance in expression level under the two conditions. The necessity of *H*_02_ for the ST was formally examined under normality setting [4]. It tends to inflate type I error rate for rejecting mean equality if the smaller sample is from the population with the larger variance. In contrast, it tends to be conservative if the larger sample is from the population with smaller variance. The WT [5] is an adaptation of the ST to allow for potential variance heterogeneity between two experimental conditions. This test calibrates potential variance heterogeneity as an impediment to identify differentially expressed genes. Demissie et al. developed the MWT [6] to obtain more stable estimates of the error variance of a gene in a low-replicate microarray experiment. The MWT outperformed the Welch test to allow for variance heterogeneity. All aforesaid tests either simply ignore or take the variance heterogeneity as an impediment and calibrate it when identifying differentially expressed genes.

In comparative microarray experiments, condition change may alter entire expression distributions of susceptible genes. Genes can interact with each other and interact with environmental factors. For a gene in a complex network, its distribution heterogeneity of expression levels can include heterogeneities in mean, variance, and even higher-order mathematical characteristics. Thus far, researchers have been conventionally focusing on exploiting mean heterogeneity, simply ignoring or adjusting for overall intra-condition variance heterogeneity. Herein, we distinguish ‘informative component’ from ‘impediment component’ of the overall variance heterogeneity. Specifically, we call the variance heterogeneity due to condition change as ‘informative variance heterogeneity’; and call variance heterogeneity due to environmental covariates and latent factors (i.e., background data structure) as impediment variance heterogeneity. However, informative variance heterogeneity has not been well recognized and exploited.

Informative variance heterogeneity of a susceptible gene can capture extra information conveyed by complicated biological networks. High gene-gene correlations are common in co-expression networks of differentially expressed genes [7, 8]. Genes can interact with each other and/or interact with environmental factors. Therefore, the alteration of expression distribution of a susceptible gene cannot be completely determined by its mean heterogeneity. Heterogeneities of high-order characteristics, e.g., variance and kurtosis, can provide extra valuable information. Exploiting informative mean heterogeneity of gene expression level alone would be incompetent to extract the information of the second-order moment (i.e., the variance). In context of genetic association studies, there are existent methods for integrating variance heterogeneity to identify genetic loci which are associated with the variances of quantitative traits (vQTL) [9, 10] and gene expression levels (evQTL) [11]. In addition, KA Geiler-Samerotte [12] presented several biological examples and also argued that variance heterogeneities of biological data may provide insight about phenotypic variability. Detecting QTLs, however, is different from detecting differential expressions between comparative microarray experiments. Existent methods cannot explicitly integrate the informative variance heterogeneity of gene expressions due to condition change; and little has been done to distill informative variance heterogeneity.

In this article, we put forth mean-variance differentially expressed (MVDE) gene as a novel concept. The family of MVDE genes is broader than that of conventional MDE genes. It goes one step closer to our generic concept of a susceptible gene − a gene displays reliable changes in any aspects of the entire distribution of its expression level with the change in condition. A MVDE gene may display different means and/or variances of expression levels between two different conditions. The proper null hypothesis of testing MVDE is *H*_03_ = *H*_01_ ∩ *H*_02_: the gene has equal mean and equal variance of expression levels between the two conditions. We reject the dual null hypothesis (*H*_03_) and claim the testing gene if the data raises significant evidence for mean heterogeneity, variance heterogeneity, or both. Under normality setting, the two-sample *F*-test is the most powerful procedure for exploiting variance heterogeneity. But the *F*-test is very sensitive to the violation of normality [13]. Beyond normality setting, the Levene test [14] and the Brown–Forsythe test [15] are two popular alternatives for inspecting variance heterogeneity.

We mathematically proved and empirically illustrated that testing statistics of mean heterogeneity and variance heterogeneity are independently distributed under *H*_03_. This null independence is not well-known to many, but is crucial to assure the type I error rate control of the IMVT using Fisher’s method [15]. Under comprehensive simulations, the IMVT appeared noticeably more powerful than existent mean heterogeneity tests (i.e., WT, MWT and STSD) as well as the LRT and the SMVT for identifying MVDE genes. In particular, the IMVT appeared strikingly more powerful than the mean heterogeneity tests to identify genes with variance heterogeneity. To illustrate the practical utility of our IMVT, we reanalyzed the gene profiles of peripheral circulating B cells [16] after adjusting for global confounders and background data structure. Our IMVT replicated previous discoveries and identified novel genes that were missed by existent mean heterogeneity tests. Our results highlighted the importance of exploiting informative variance heterogeneity, which is a rich resource about the biology mechanism of gene expressions.

## Methods

Let the dataset contain expression levels of *M* gene probes of *n*_*c*_ unrelated subjects from condition *c* (i.e., *c* = 1 for control group, and *c* = 2 for treatment group). To be specific, let *G*_*ijc*_ be the expression level of gene probe *i* (=1,2,…,*M*) on subject *j* (=1,2,…,*n*_*c*_) under condition *c*, and let *n* = *n*_1_ + *n*_2_ be the total sample size. Let *μ*_*ic*_ and *σ*_*ic*_^2^ be the gene-specific mean and variance of the expression levels of gene probe *i* under condition *c*, respectively. The standard unbiased estimators of *μ*_*ic*_ and *σ*_*ic*_^2^ are given by ${\widehat{\mu}}_{ic}={\overline{G}}_{ic}={\displaystyle {\sum}_{j=1}^{n_c}}{G}_{ijc}/{n}_c$ and ${\widehat{\sigma}}_{ic}^2={\displaystyle {\sum}_{j=1}^{n_c}}{\left({G}_{ijc}-{\overline{G}}_{ic}\right)}^2/\left({n}_c-1\right)$, respectively.

### Concept of MDE genes and mean heterogeneity tests

Researchers conventionally focus on identifying MDE genes. A MDE gene displays mean differentials between the expression levels under two experimental conditions (*μ*_1_ ≠ *μ*_2_). The ST has been widely used routine to identify MDE genes. This mean heterogeneity test rejects the null hypothesis *H*_01_ : *μ*_1_ = *μ*_2_ if the Student statistic of the testing gene departures from zero significantly. A default assumption behind the ST is variance equality *H*_02_ : *σ*_1_^2^ = *σ*_2_^2^ at the testing gene. Specifically, for the *i* ^*th*^ gene, let ${\boldsymbol{G}}_1={\left({G}_{i11},{G}_{i21},\dots, {G}_{i{ n}_11}\right)}^{\prime }$ and ${\boldsymbol{G}}_2={\left({G}_{i12},{G}_{i22},\dots, {G}_{i{ n}_22}\right)}^{\prime }$ be the expression levels of two independent random samples from normal populations $\mathcal{N}\left({\mu}_{i1},{\sigma_i}_1^2\right)$ and $\mathcal{N}\left({\mu}_{i2},{\sigma}_{i2}^2\right)$, respectively. The ST on *H*_01_^(*i*)^ : *μ*_*i*1_ = *μ*_*i*2_ assumes variance homogeneity (*H*_02_^(*i*)^ : *σ*_*i*1_^2^ = *σ*_*i*2_^2^) between the two conditions, and defines the test statistic as

$$ \widehat{t}=\frac{{\left(\frac{1}{n_1}+{\frac{1}{n}}_2\right)}^{-\frac{1}{2}}\left({\widehat{\mu}}_{i1}-{\widehat{\mu}}_{i2}\right)}{\sqrt{{\widehat{\sigma}}_p^2}}, $$

where ${\widehat{\sigma}}_p^2=\frac{n_1-1}{n_1+{n}_2-2}{\widehat{\sigma}}_{i1}^2+\frac{n_2-1}{n_1+{n}_2-2}{\widehat{\sigma}}_{i2}^2$ is the pooled sample variance estimator of the common variance σ^2^. If *H*_03_^(*i*)^ = *H*_01_^(*i*)^ ∩ *H*_02_^(*i*)^ is true, then the testing statistic $\widehat{t}$ follows the centralized Student *t* distribution with (*n*_1_ + *n*_2_ − 2) degrees of freedom $\left(\widehat{t}\sim {t}_{n_1+{n}_2-2}\right)$. It is well known that violating the assumption of variance homogeneity would result in type I error inflation or power loss of the ST [17].

The WT, as an adaptation of the ST, is more reliable when the two-group samples have unequal variances and unequal sample sizes. The Welch statistic is defined by

$$ \widehat{WT}=\frac{{\widehat{\mu}}_{i1}-{\widehat{\mu}}_{i2}}{\sqrt{\frac{{\widehat{\sigma}}_{i1}^2}{n_1}+\frac{{\widehat{\sigma}}_{i2}^2}{n_2}}} $$

This statistic calibrates the impact of potential variance heterogeneity between two conditions. For a gene with equal means between two conditions (regardless of variance heterogeneity), $\widehat{WT}$ approximately follows a *t*-distribution with the following Welch-Satterthwaite degree of freedom:

$$ \nu =\frac{{\left(\frac{{\widehat{\sigma}}_{i1}^2}{n_1}+\frac{{\widehat{\sigma}}_{i2}^2}{n_2}\right)}^2}{\left(\frac{{\widehat{\sigma}}_{i1}^4}{n_1^2\left({n}_1-1\right)}+\frac{{\widehat{\sigma}}_{i2}^4}{n_2^2\left({n}_2-1\right)}\right)}. $$

To calibrate unequal variances, another alternative is the MWT [6], which would yield reliable condition-specific variance estimators for low-replicate experiments. For large-sample experiments, one can perform Student t test on standardized data (STSD), where the gene expression levels are divided by condition-specific sample standard deviations respectively.

### Concept of MVDE genes and variance heterogeneity tests

A gene is called to be susceptible if the change in condition can alter arbitrary aspects of the entire distribution of its expression level, i.e., mean, variance, kurtosis and/or even higher-order characteristics. The term MVDE gene is adopted to describe a gene whose mean and/or variance in expression level is sensitive to the change in condition. Formally, a MVDE gene has different means (*μ*_1_ ≠ *μ*_2_) and/or variances (*σ*_1_^2^ ≠ *σ*_2_^2^) of expression levels between two conditions. This concept of MVDE genes goes one step closer to our general concept of a susceptible gene and is more reasonable than the conventional concept of MDE genes, which confines to differential mean expression levels only. In gene co-expression networks, genes work together and the expression levels are correlated. Some susceptible genes may also interact with other susceptible genes and/or environmental factors. Such correlations and interactions among biological networks are very common and are major drivers for the variance heterogeneity of a test susceptible gene. Variance heterogeneity, to some extent, indicates how a gene involve in complex networks. Therefore, we argue that variance heterogeneity should be as equally important as mean heterogeneity for identifying differentially expressed genes. To identify susceptible genes, one crucial step is to extract summary statistics containing potential information about variance heterogeneity, i.e., the *p* values computed from some appropriate test statistic on the null hypothesis *H*_02_^(*i*)^ (variance homogeneity).

For a random gene, if its (transformed) expression levels follow normal distribution, then the classical two-sample *F*-statistic

$$ \widehat{F}=\frac{{\widehat{\sigma}}_{i1}^2}{{\widehat{\sigma}}_{i2}^2} $$

follows the centralized *F*-distribution with (*n*_1_ − 1) and (*n*_2_ − 1) degrees of freedom $\left(\widehat{F}\sim {F}_{n_1-1,{n}_2-1}\right)$ since *H*_02_^(*i*)^ is true. Under normality setting, the *F*-test is the most powerful test for exploiting variance heterogeneity. Nevertheless, the *F*-test is very sensitive to the violation of normality. Therefore, it may claim random genes to be spuriously significant if their (transformed) expression levels do not strictly follow normal distributions. Actually, the two-sample *F* test is more suitable for testing normality other than variance heterogeneity [13].

As a robust alternative, the Brown-Forsythe statistic is the *F*-ratio that stems from applying the ordinary one-way analysis of variance on the absolute deviations from the median:

$$ \widehat{BF}=\frac{\left({n}_1+{n}_2-2\right){\displaystyle {\sum}_{c=1}^2}{n}_c{\left({\overline{Z}}_{i c}-{\overline{Z}}_i\right)}^2}{{\displaystyle {\sum}_{c=1}^2}{\displaystyle {\sum}_{j=1}^{n_c}}{\left({Z}_{i jc}-{\overline{Z}}_{i c}\right)}^2}, $$

where ${Z}_{ijc}=\left|{G}_{ijc}-{\tilde{G}}_{ic}\right|$, ${\overline{Z}}_{ic}=\frac{1}{n_c}{\displaystyle {\sum}_{j=1}^{n_c}}{Z}_{ijc}$, ${\overline{Z}}_i=\frac{1}{n_1+{n}_2}{\displaystyle {\sum}_{c=1}^2}{\displaystyle {\sum}_{j=1}^{n_c}}{Z}_{i jc}$, and ${\tilde{G}}_{ic}= median\left({\boldsymbol{G}}_{\boldsymbol{c}}\right)$. When *H*_02_^(*i*)^ is true, the distribution of $\widehat{BF}$ follows approximately the *F*-distribution with degrees of freedom 1 and (*n*_1_ + *n*_2_ − 2).

Another alternative, the Levene test, uses the mean instead of the median:

$$ \widehat{LF}=\frac{\left({n}_1+{n}_2-2\right){\displaystyle {\sum}_{c=1}^2}{n}_c{\left({\overline{Z}}_{i c}-{\overline{Z}}_i\right)}^2}{{\displaystyle {\sum}_{c=1}^2}{\displaystyle {\sum}_{j=1}^{n_c}}{\left({Z}_{i jc}-{\overline{Z}}_{i c}\right)}^2}, $$

where ${Z}_{i jc}=\left|{G}_{i jc}-{\overline{G}}_{i c}\right|,{\overline{Z}}_{i c}=\frac{1}{n_c}{\displaystyle {\sum}_{j=1}^{n_c}}{Z}_{i jc},{\overline{Z}}_i=\frac{1}{n_1+{n}_2}{\displaystyle {\sum}_{c=1}^2}{\displaystyle {\sum}_{j=1}^{n_c}}{Z}_{i jc}$ and ${\overline{G}}_{ic}= mean\left({\boldsymbol{G}}_{\boldsymbol{c}}\right)$. If *H*_02_^(*i*)^ is true, then $\widehat{LF}$ follows approximately the *F* distribution with degrees of freedom 1 and (*n*_1_ + *n*_2_ − 2).

For each gene, the optimal test for variance heterogeneity depends on the underlying gene expression distribution. According to Brown and Forsythe’s Monte Carlo studies [15], the Levene test provided the best power for symmetric, moderate-tailed distributions; whereas the Brown-Forsythe test performed best when the underlying data followed heavily skewed distributions.

### Integrating mean and variance heterogeneities

One most commonly used method to integrate two independent pieces of information is Fisher’s linear combination. For a testing gene, let *p*_*WT*_, *p*_*F*_, *p*_*BF*_, *p*_*LF*_ denote the *p*-values of the Welch statistic, the *F* statistic, the Brown-Forsythe statistic and the Levene statistic, respectively. We recommend using $\widehat{IMVT}=-2\left( \log \left({p}_{WT}\right)+ \log \left({p}_{LF}\right)\right)$ to integrate mean and variance heterogeneities. Another two alternatives are $\widehat{ F WT}=-2\left( \log \left({p}_{WT}\right)+ \log \left({p}_F\right)\right)$ and $\widehat{ BF WT}=-2\left( \log \left({p}_{WT}\right)+ \log \left({p}_{BF}\right)\right)$. Each of the three Fisher linear combinations follows approximately the *χ* ^2^ - distribution with 4° of freedom, provided that the *p*-values of mean heterogeneity tests are independent of the *p*-values of variance heterogeneity tests under joint null *H*_03_.

### Alternative tests for the joint null hypothesis of mean and variance equalities

To test *H*_03_, a framework of separate mean and variance tests (SMVT) can also be conducted. This framework applies WT on *H*_01_ (mean equality) at nominal level *α*_1_ and Levene test on *H*_02_ (variance equality) at nominal level *α*_2_, respectively. *H*_03_ is rejected if *H*_01_ or *H*_02_ or both are rejected. By our proposition on the null independence, type I error rate of this framework is given by *α* = *α*_1_ + *α*_2_ − *α*_1_ *α*_2_. It is intractable to choose universal optimal *α*_1_ and *α*_2_ for all genes. To control the overall type I error rate at nominal level *α*, one typical choice is setting ${\alpha}_1={\alpha}_2=1-\sqrt{\alpha}$. Similar as Fisher’s linear combination, the SMVT gives equal weight to mean heterogeneity and variance heterogeneity.

The two-sample *LRT* is another alternative to test *H*_03_, assuming the (transformed) expression levels follow normal distributions. Specifically, the LRT statistic is given by

$$ \widehat{LRT}=\frac{{\left(\frac{n_1-1}{n_1}{\widehat{\sigma}}_{i1}^2\right)}^{\frac{n_1}{2}}{\left(\frac{n_2-1}{n_2}{\widehat{\sigma}}_{i2}^2\right)}^{\frac{n_2}{2}}}{{\left(\frac{1}{n_1+{n}_2}\left({\displaystyle {\sum}_{j=1}^{n_1}}{\left({G}_{i j1}-\widehat{\mu}\right)}^2+{\displaystyle {\sum}_{j=1}^{n_2}}{\left({G}_{i j2}-\widehat{\mu}\right)}^2\right)\right)}^{\frac{n_1+{n}_2}{2}}}, $$

$\widehat{\mu}=\frac{1}{n_1+{n}_2}\left({\displaystyle {\sum}_{j=1}^{n_1}}{G}_{ij1}+{\displaystyle {\sum}_{j=1}^{n_2}}{G}_{ij2}\right)$ (See the Additional file 1 for mathematical derivation of the LRT statistic). Under normal setting with *H*_03_, ${\widehat{\chi}}_2^2=-2 \ln \left(\widehat{LRT}\right)$ follows *χ* ^2^ - distribution with 2° of freedom asymptotically for large sample sizes.

## Results

### The null independence between the mean and variance heterogeneity tests

It’s commonly believed that testing statistics of mean and variance heterogeneities are dependently distributed, even if the data forming them are from an identical normal population. Actually, this is a widespread misunderstanding due to the forms of the testing statistics. For example, both Student’s *t*-statistic and the *F*-statistic are defined in terms of sample variances. In fact, all aforesaid testing statistics of mean heterogeneity are independent of all aforesaid testing statistics of variance heterogeneity under *H*_03_. This null independence lays the foundation of type I error rate control of the integrative heterogeneity tests. Herein, we formally formulate the finite-sample null independence by the following proposition.

**Proposition**: *Student t statistic and Welch t statistic are independent of the F-, Levene and Brown-Forsythe statistics if the finite samples (* ***G***_*1*_ *,* ***G***_*2*_ *) forming them jointly follow an arbitrary spherically symmetric distribution.*

The proposition formulates the finite-sample null independence under a broader distribution family, including normality as a special member (for mathematical proofs, see Additional file 2: Appendixes A and B). Its typical members include multivariate Gaussian, Student, Kotz, exponential power, Laplace distributions with spherically symmetric variance-covariance matrices [13]. Many researchers are familiar with and usually adopt normality assumption on (transformed) gene expression levels. By this proposition, if the normality assumption is met, the proposed integrative heterogeneity tests can properly control the type I error rate. However, the normality assumption is often violated more or less by real-world gene expression data. Rigorously speaking, no transformation of gene expression data can assure exact normality. Therefore, it is necessary and useful to extend the null independence to broader distribution families, e.g., spherically symmetric family.

### Empirical illustrations of the proposition

**Fig. 1:**
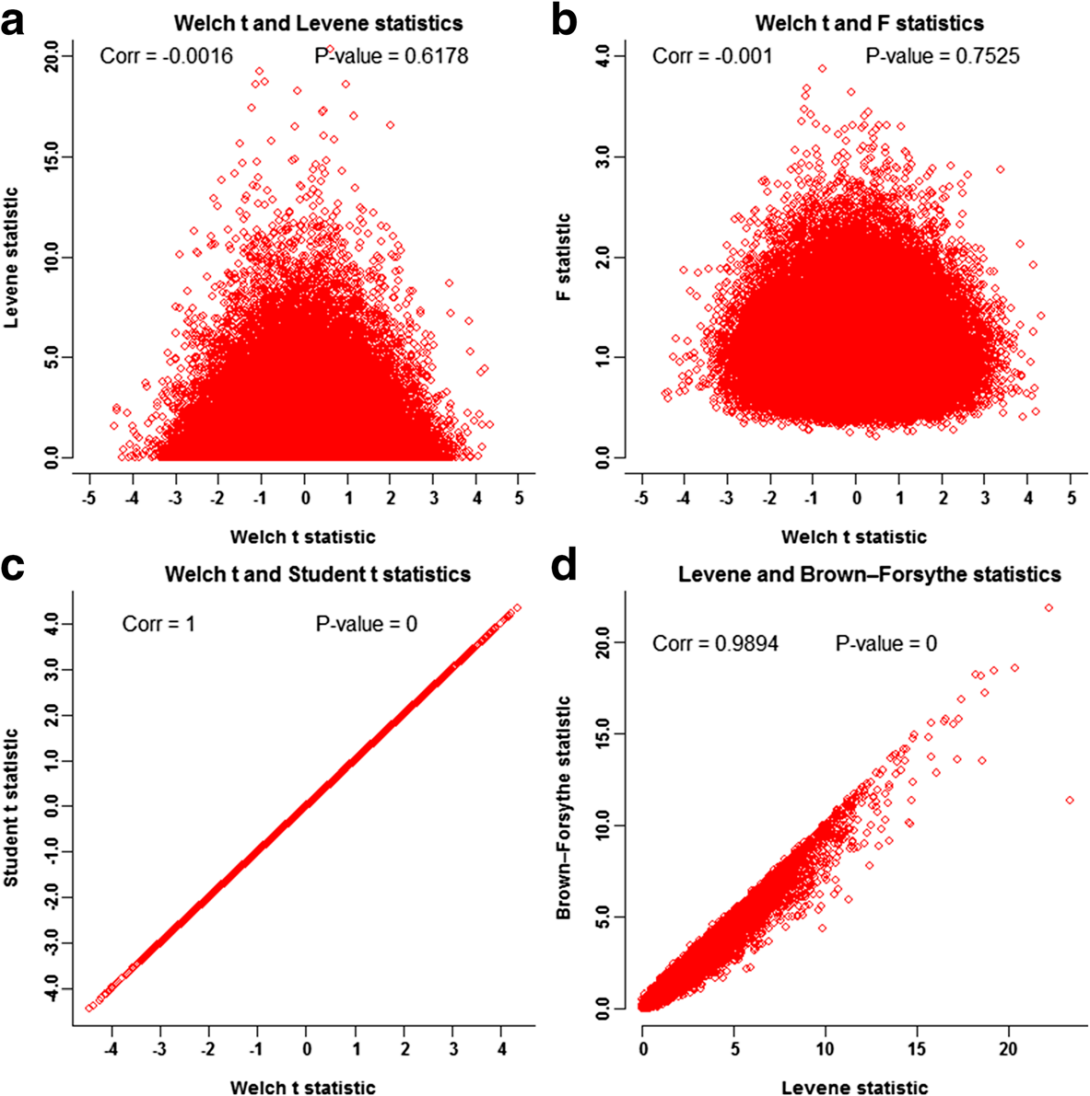
Null joint distributions of the test statistics on mean and variance heterogeneities under normality setting. Each panels displays 100,000 pairs of the specified test statistics, which were computed from 100,000 replicates of two-group samples of sizes (*n*_1_ = *n*_2_ = 40) from the standard normal distribution. Panel **a** shows the null independence between Welch *t* statistic and Levene statistic. Panel **b** shows the null independence between Welch *t*-statistic and *F*-statistic. Panel **c** shows the equivalence between Welch *t* statistic and Student t statistic. Panel **d** shows the high correlation between Levene test statistic and Brown-Forsythe statistic

First, we generated 100,000 replicates of two-group samples from the standard normal distribution with sample size *n*_1_ = *n*_2_ = 40. As anticipated by the proposition, the vast majority of replicate-specific pairs of Welch t statistic $\left(\widehat{WT}\right)$ and Levene statistic $\left(\widehat{LF}\right)$ randomly concentrates around (0, 1) (Fig. 1a) and so do the replicate-specific Welch t statistic and *F* statistic pairs (Fig. 1b). Under this simulation design, Welch t and Student t statistics $\left(\widehat{WT},\widehat{t}\right)$ appeared equivalent (Fig. 1c). The correlation between Levene statistic $\left(\widehat{LF}\right)$ and Brown-Forsythe statistic $\left(\widehat{BF}\right)$ turned to be 0.9894 (Fig. 1d). The scatterplots of $\left(\widehat{t},\widehat{LF}\right)$, $\left(\widehat{t},\widehat{BF}\right)$, and $\left(\widehat{t},\widehat{F}\right)$ are qualitatively the same as those of $\left(\widehat{WT},\widehat{LF}\right)$ (Results not shown here). Under the normality setting with smaller sample sizes, we also obtained the corresponding figures for some other sample sizes (Additional file 2: Figure S1.1–Figure S1.3, Appendix C), which revealed very similar patterns to Fig. 1. Standard multi-variate normal distribution is a typical member in the family of spherically symmetric distributions. These simulation results illustrate the null independence within the family of all spherically symmetric distributions.

As explorations outside of the spherically symmetric family, we performed comprehensive simulations by generating the data from the standard Laplace distribution. Univariate Laplace distribution is a typical member of the family of symmetric distributions. However, the joint distribution of independent univariate Laplace variables is outside of the spherically symmetric distribution family. Under the standard Laplace setting, we obtained the corresponding scatterplots and observed similar patterns of the joint distributions of the mean and variance test statistics (Additional file 2: Figure S2.1–Figure S2.4, Appendix C). These empirical results illustrate the robustness of the null independence between mean and variance tests for the data from the family of symmetric distributions.

### Type I error rates control of the competitors

**Fig. 2:**
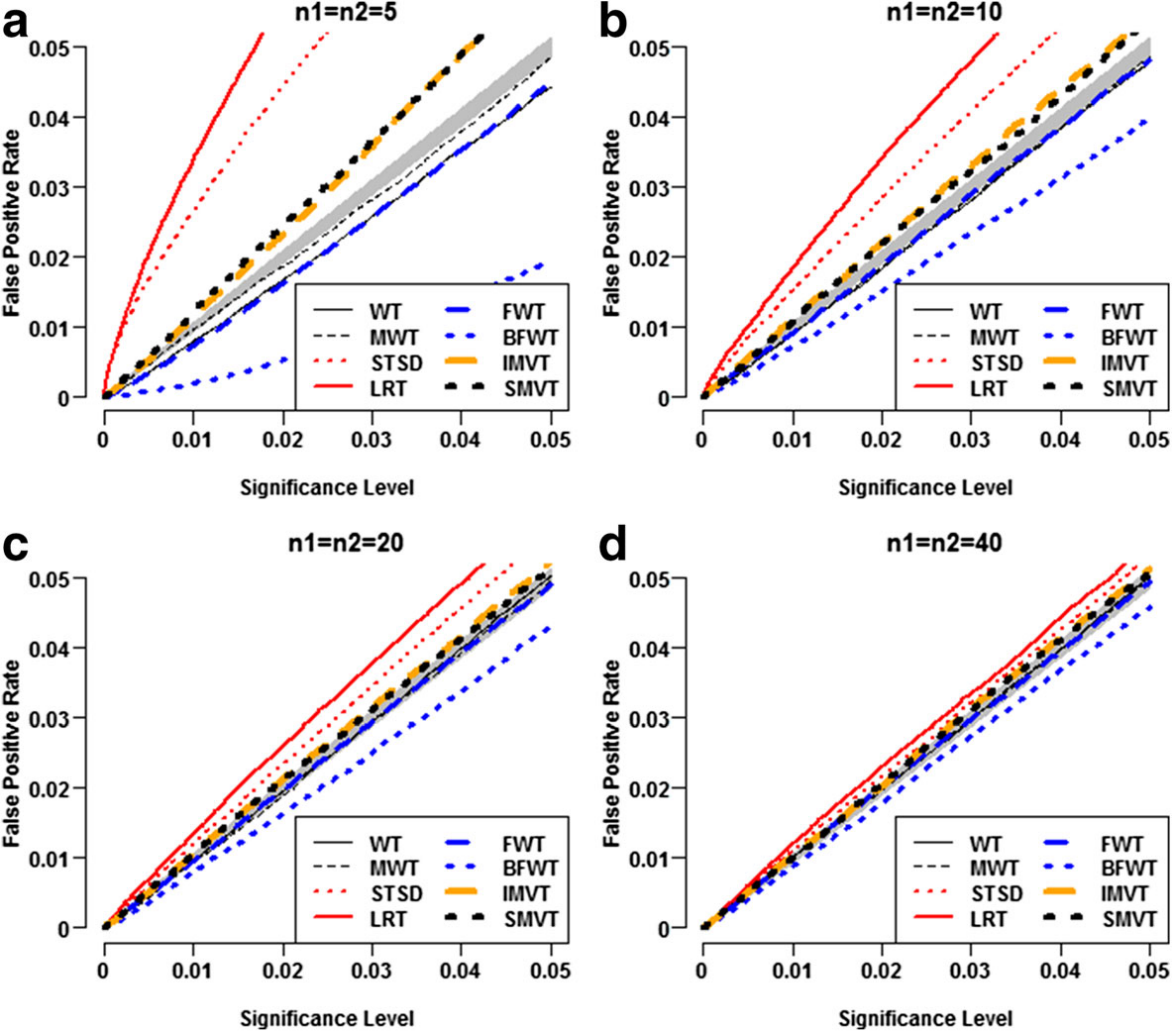
Comparison of false positive rates of eight methods under standard normality setting. Each panel was computed from 100,000 replicates of two-group samples with the specified samples sizes simulated from $\mathcal{N}\left(0,1\right)$. At each significance level, the false positive rate of each method was estimated by the empirical proportion that the method rejected the dual null hypothesis H_03_. The gray belt is the 95% concentration band of the false positive rates of a typical test that can properly control false positive rates at given nominal significance levels. **a** n1=n2=5, **b** n1=n2=10, **c** n1=n2=20, **d** n1=n2=40

Under normality setting. With extremely small samples, none of the eight competitors could properly control type I error rates (Fig. 2a). The LRT and the STSD severely inflated type I error rates. The IMVT and the SMVT appeared equally anti-conservative; both were much less anti-conservative than the LRT and the STSD. The MWT performed the best to control type I error rates; it was slightly conservative. The WT and the FWT appeared equally conservative; both were clearly more conservative than the MWT. The BFWT appeared severely conservative. The LRT inflated the type I error rates because the *χ*_2_^2^ distribution could not well approximate the exact distribution of the LRT statistic. The anti-conservative of the STSD stemmed from the variability of condition-specific data standardization. Specifically, sample standard deviations of small samples could not precisely estimate the standard deviation. The conservativeness of the BFWT stemmed from the well-known conservativeness of the Brown-Forsythe test [18, 19]. For larger sample sizes (Fig. 2b-d), the LRT, the STSD, the SMVT and the IMVT appeared less anti-conservative, and the MWT, the WT, the FWT and the BFWT became less conservative. When sample sizes reached 40, the IMVT and the SMVT as well as the WT, the MWT and the FWT properly controlled the Type I error rates (Fig. 2d).

**Fig. 3:**
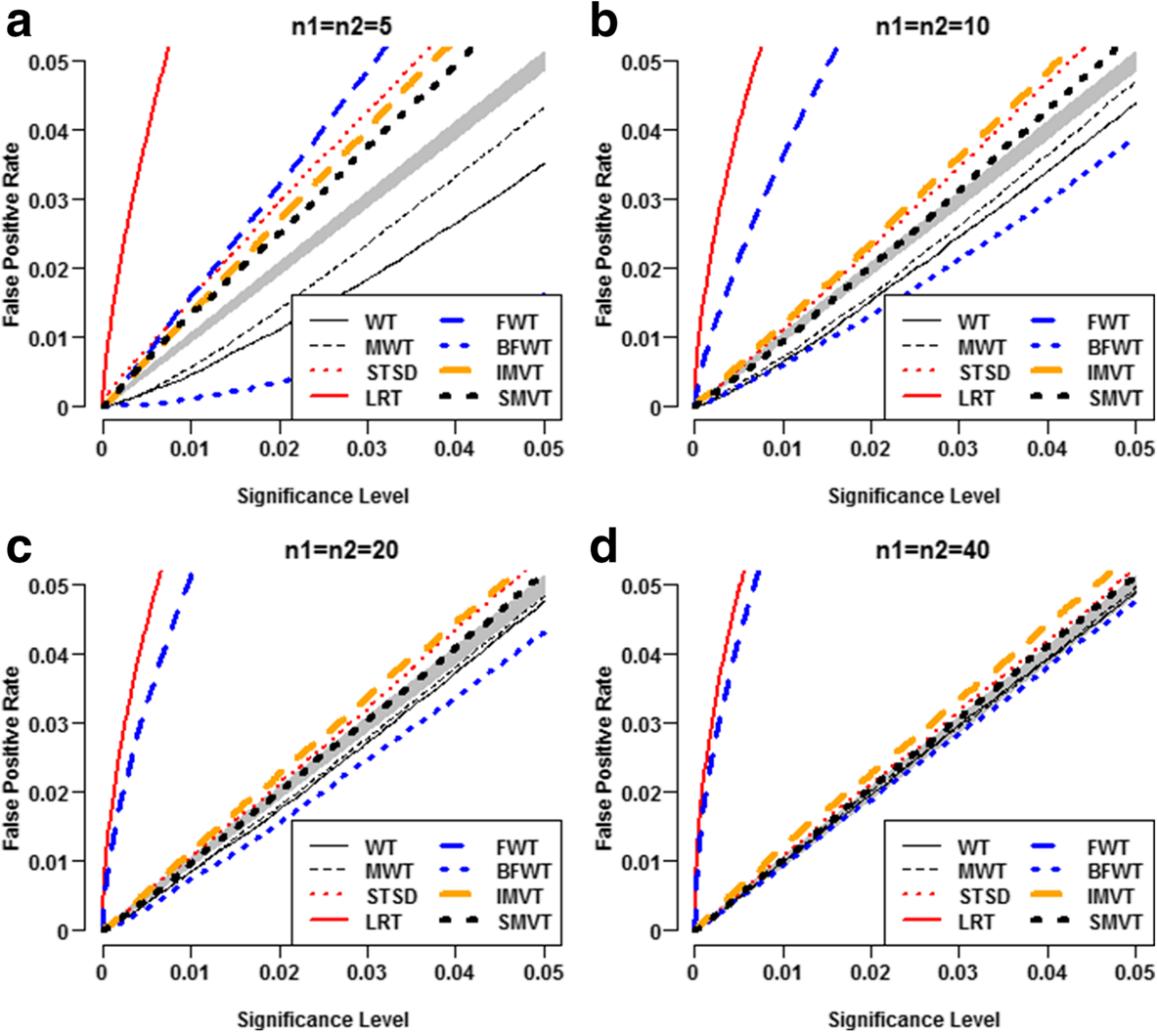
Comparison of false positive rates of eight methods under standard Laplace setting. Each panel was computed from 100,000 replicates of two-group samples with the specified samples sizes simulated from ℒ*aplace*(0, 1). At each significance level, the false positive rate of each method was estimated by the empirical proportion that the method rejected the dual null hypothesis H_03_. The gray belt is the 95% concentration band of the false positive rates of a typical test that can properly control false positive rates at given nominal significance levels. **a** n1=n2=5, **b** n1=n2=10, **c** n1=n2=20, **d** n1=n2=40

Under the Laplace setting, the LRT and the FWT appeared severely anti-conservative (Fig. 3a-d). Their inflations in type I error rate appeared even severer as the samples increased. The LRT had inflated type I error rates because it was derived from normality assumption of gene expression levels. The FWT had inflated type I error rates because the *F* test statistic is very sensitive to the non-normality of the samples [13]. The other tests displayed similar patterns to those under normality setting. For extremely small sample sizes, the STSD, the IMVT and the SMVT appeared successively less anti-conservative; whereas the MWT, the WT and the BFWT appeared successively more conservative (Fig. 3a). Their magnitudes of inflations and deflations in type I error rate appeared to vanish as the sample sizes increased (Fig. 3b-d).

### Empirical power comparisons under normality setting and non-normality setting

For power comparisons, we investigated three kinds of scenarios under both normality setting and Laplace setting: (1) unequal mean and equal variance, (2) equal mean and unequal variance and, (3) unequal mean and unequal variance. For sample sizes as large as *n*_1_ = *n*_2_ *=* 40, the proposed and existent tests well controlled type I error rates under normality and Laplace setting. And the sample size is very close to those of the gene expression files of Pan et al. [16]. We thus presented here the power comparisons with the sample sizes *n*_1_ = *n*_2_ *=* 40.

**Fig. 4:**
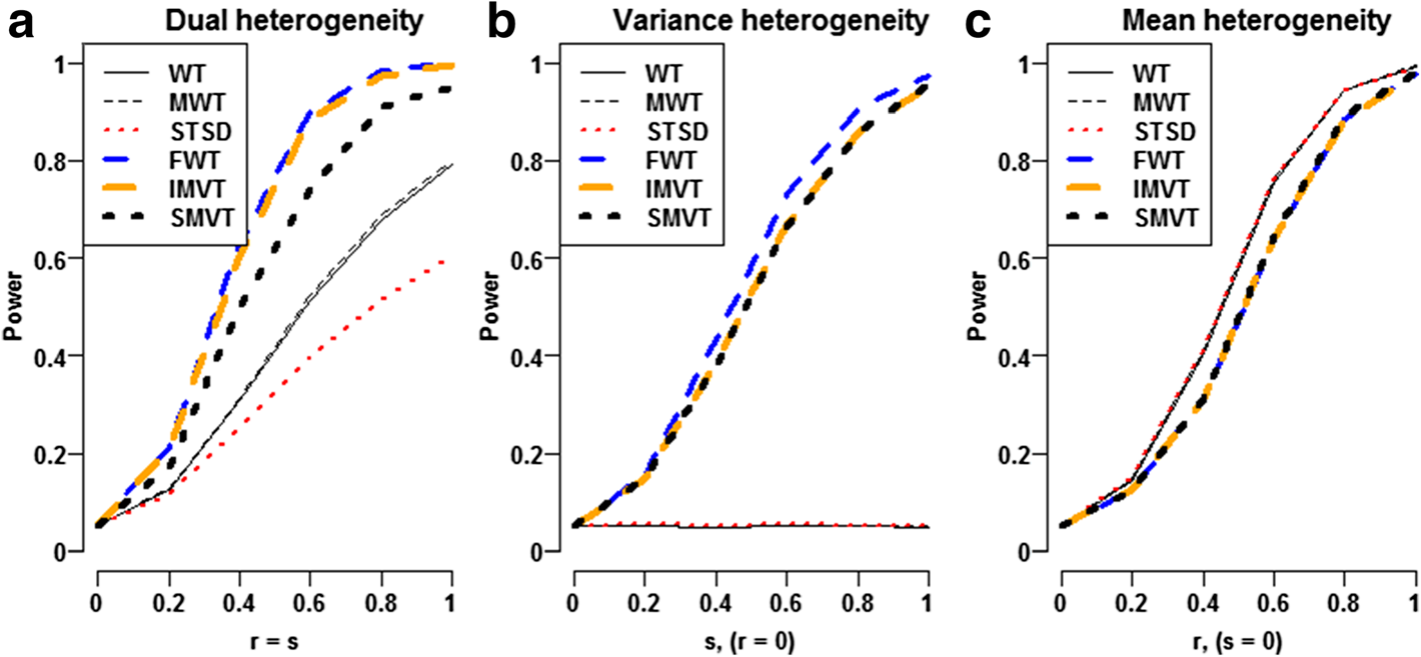
Power comparison of six methods under two-condition normality setting. In each panel, for each specific (r, s) pair, powers of the six methods were computed from 100,000 replicates of two-group samples with samples sizes (40 vs. 40) simulated from $\mathcal{N}\left(0,1\right)$ and $\mathcal{N}\left( r,{\left(1+ s\right)}^2\right)$, respectively. At each (r, s) pair, the power of each method was estimated by the empirical proportion that the method rejected the dual null hypothesis H_03_ at significance level 0.05. For the SMVT, both the significance level of Welch test and that of the Levene test were set to be $1-\sqrt{1-0.05}$ to control overall type I error rate at 0.05. **a** Dual heterogeneity, **b** Variance heterogeneity, **c** Mean heterogeneity

Under normality setting, we simulated independently 10,000 replicates of *n*_1_ = 40 data points from normal distribution $\mathcal{N}\left(0,1\right)$ and *n*_2_ = 40 data points from $\mathcal{N}\left( r,{\left(1+ s\right)}^2\right)$ for each (*r*, *s*) pair. Herein, the parameters *r* and *s* represent the magnitudes of mean and variance heterogeneities, respectively. When *s* ≠ 0, the IMVT and the FWT displayed the highest powers, followed by the SMVT; and all the three joint heterogeneity tests outperformed the three mean heterogeneity tests, i.e., the WT, the MWT and the STSD (Fig. 4a-b). The power gains of the joint heterogeneity tests over the mean heterogeneity tests appeared especially noteworthy when *s* ≠ 0 and *r* = 0 (Fig. 4b). The joint heterogeneity tests did not display severe power losses even for the theoretical scenarios favoring the mean tests (Fig. 4c). In addition, the FWT slightly outperformed the IMVT because the F test statistic is the optimal test statistic for variance heterogeneity under normality setting. Here, we did not compare the powers of the LRT and the BFWT since they could not control type I error rates.

**Fig. 5:**
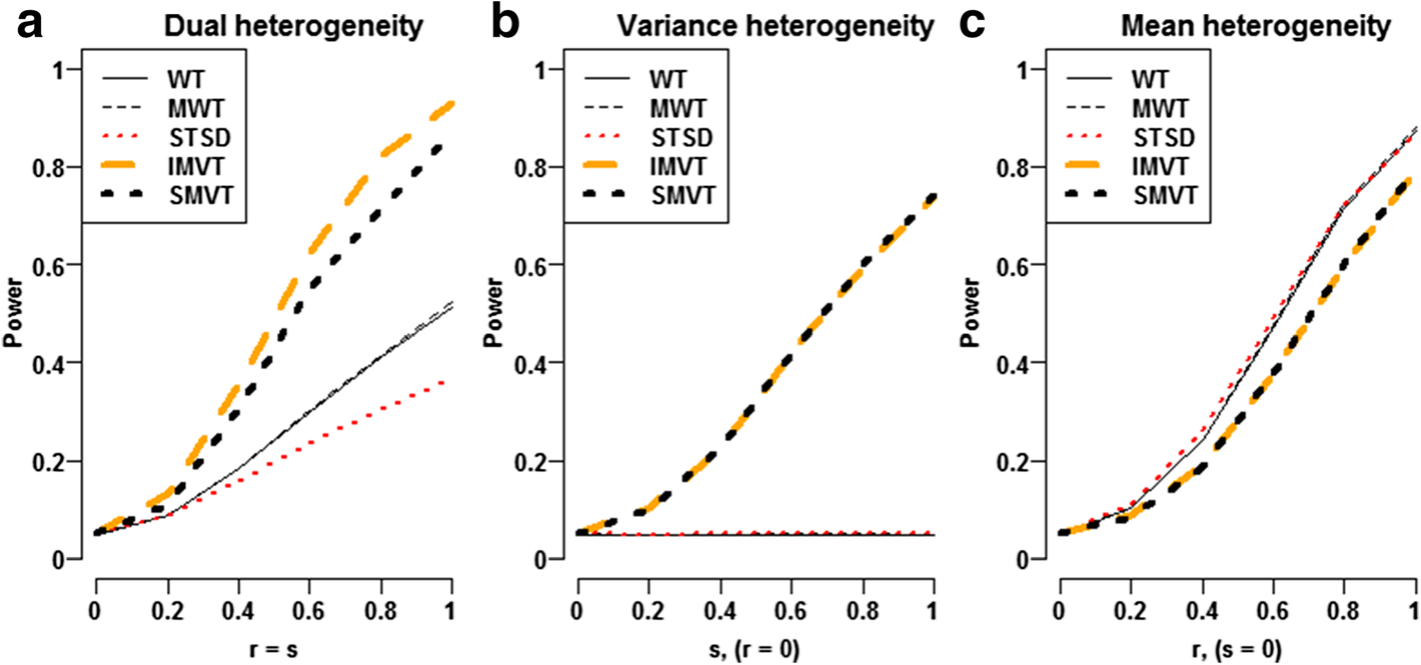
Power comparison of six methods under two-condition Laplace setting. In each panel, for each specific (r, s) pair, powers of the six methods were computed from 100,000 replicates of two-group samples with samples sizes (40 vs. 40) simulated from ℒ*aplace*(0, 1) and ℒ*aplace*(*r*, (1 + *s*)^2^), respectively. At each (r, s) pair, the power of each method was estimated by the empirical proportion that the method rejected the dual null hypothesis H_03_ at significance level 0.05. For the SMVT, both the significance level of Welch test and that of the Levene test were set to be $1-\sqrt{1-0.05}$ to control overall type I error rate at 0.05. **a** Dual heterogeneity, **b** Variance heterogeneity, **c** Mean heterogeneity

Under Laplace setting, we simulated independently 10,000 replicates of *n*_1_ = 40 data points from standard Laplace distribution *Laplace* (0, 1) and *n*_2_ = 40 data points from *Laplace* (*r*, (1 + *s*)^2^) for each (*r*,*s*) pair. Again, the parameters *r* and *s* represent the magnitudes of mean and variance heterogeneities, respectively. Under the Laplace setting, we observed qualitatively the same patterns as those under the normality setting. When *s* ≠ 0, the IMVT outperformed the SMVT; and both the joint heterogeneity tests outperformed the three mean heterogeneity tests, i.e., the WT, the MWT and the STSD (Fig. 5a-b). The power gains of the joint heterogeneity tests over the mean heterogeneity tests appeared especially noteworthy when *s* ≠ 0 and *r* = 0 (Fig. 5b). The joint heterogeneity tests did not display severe power losses even for the theoretical scenarios favoring the mean heterogeneity tests (Fig. 5c). Here, we did not compare the powers of the LRT, the FWT and the BFWT since they could not control type I error rates under non-normality setting.

These results formally demonstrate the importance of integrating informative variance heterogeneity. In general, the power gains of the IMVT over its competitors are solid. For the scenarios of mean heterogeneity only, the IMVT would have small power losses. All in all, the IMVT displayed valuable merits over its competitors. At least, the IMVT is an admissible procedure. It should be useful to improve the power to identify susceptible genes involved in co-expression networks. By its robustness to non-normality data, we recommend the IMVT as a powerful alternative to exploit microarray profiles.

### Re-analyzing the gene expression profiles of peripheral circulating B Lymphocytes

**Fig. 6:**
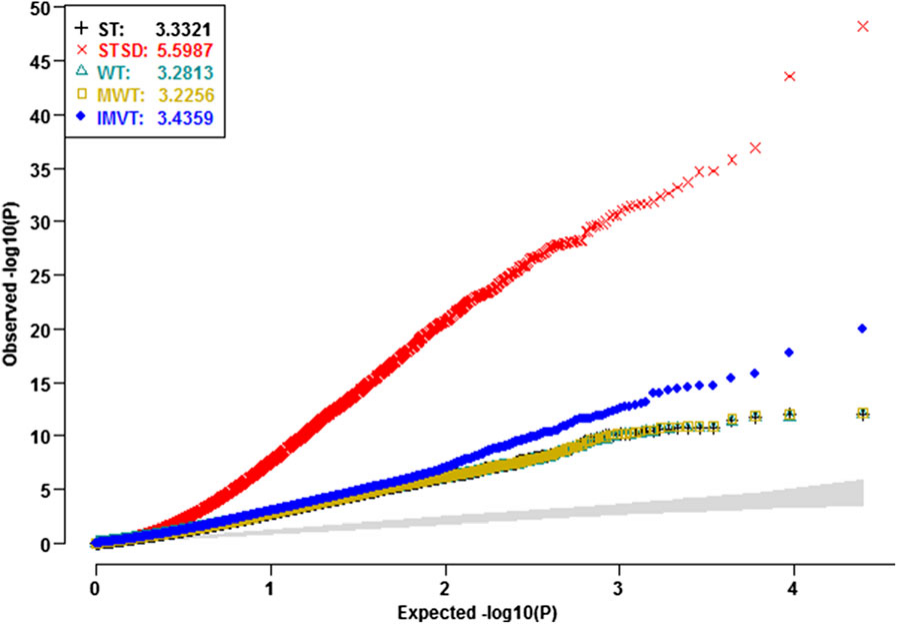
*Q-Q* plots of the five competitors without adjusting for latent data structure and covariates. Using the MAS5, we normalized the raw expression data of the 22,283 gene probes on the 39 smokers and 40 nonsmokers. We then compute gene probe specific statistics and *p* values of the tests statistics based on the MAS5 normalized data. The inflation factors of all the tests appeared unreasonably huge, especially that of the STSD. All the curves clearly appeared above the gray band (95% concentration band). The striking inflations implied that some latent factors severely confounded the competitors

Pan et al. [16] compared the gene expressions profiles of peripheral circulating B cells between 39 smoking and 40 non-smoking healthy US white women. Using MAS5 software, they normalized the expression levels of 7215 selected probes out of all the 22,283 experiment-wide probes. They applied traditional *t* tests to the normalized expression levels and report 125 promising DE genes. The authors justified why they did not adjust for menopausal status and age. However, they neglected the latent background data structure. Using the MAS5 software, we normalized the raw expression levels of all the 22,283 experiment-wide gene probes. For the normalized data, we computed the probe specific test statistics and *p* values of five competitors. The genomic inflation factors [20] of these heterogeneity tests would be close to 1 if they could properly control type I error rates. However, all the tests displayed huge genomic inflation factors, especially the STSD (Fig. 6). All the *Q-Q* plots climbed quickly above the upper limit of the 95% concentration band (the gray band). The severe genomic inflations indicated that some major latent factors would confound all the competitors. Thus, the *t* tests performed by Pan et al. [16] would be confounded since they did not adjust for any background factors.

**Fig. 7:**
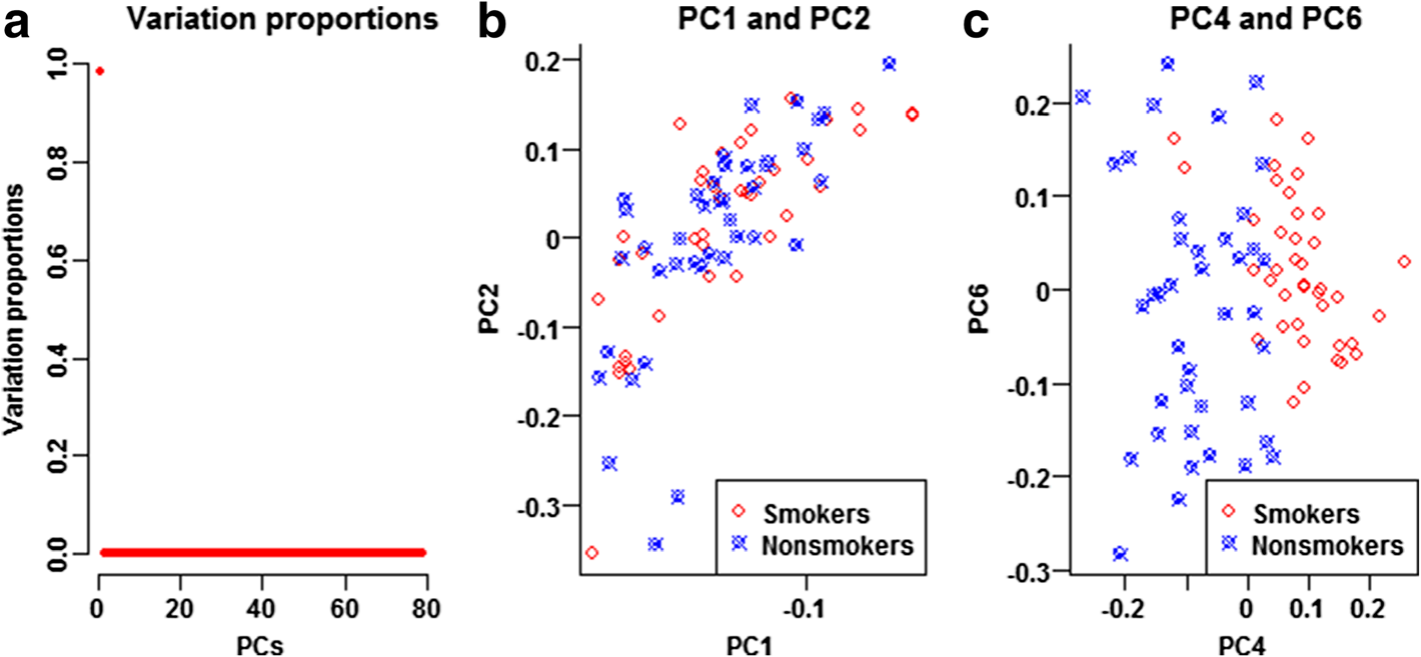
Global data structure of all the experiment-wide gene expression levels. Using MAS5, we normalized the raw expression levels of the 22,283 experiment-wide gene probes and computed the PCs of all the normalized expression levels. PC1 alone accounted for 98.24% of the total variation and was the unique major PC. PC2 merely accounted for 0.32% of total variation. Neither PC1 nor PC2 displayed mean heterogeneity or variance heterogeneity. PC4 displayed strikingly significant mean heterogeneity (*p*_*WT*_ = 1.91 × 10^− 15^), even if it only accounted for 0.13% of the total variation. PC6 displayed very significant variance heterogeneity (*p*_*LF*_ = 3.18 × 10^− 4^) even if it accounted for 0.07% of the total variation only. PC4 and PC6 clearly distinguished the smokers and the nonsmokers. **a** Variation proportions, **b** PC1 and PC2, **c** PC4 and PC6

**Fig. 8:**
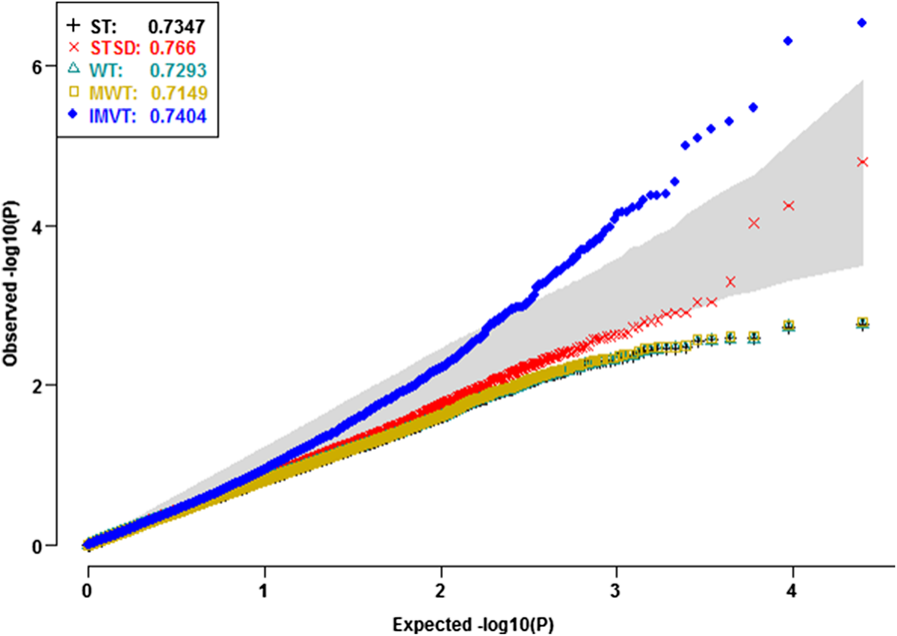
Deflations due to the over adjustment of the experiment-wide data structure. Among all the 79 global PCs, only PC4 displayed significant mean heterogeneity (*p*_*WT*_ =4.49E-15). PC6, 9, 14, 28, 38, 49 and 78 displayed variance heterogeneity (*p*_*LF*_ ranged from 3.18E-4 to 0.0419). After adjusting for the significant global PCs, age and menopausal status, the *Q-Q* plots of all the five competitors displayed severe deflations. All the genomic inflation factors turned out to be much smaller than 1. The *Q-Q* plots of the four mean heterogeneity tests fell below the diagonal, where those of the WT and the MWT fell below the lower limit of the 95% concentration band. Global PCs did not distinguish informative heterogeneities and impediment heterogeneities. The significant global PCs would account for big portions of informative mean and variance heterogeneities due to DE genes. Therefore, adjusting for the significant PCs of all the experiment-wide gene probes would reduce statistical powers

To reveal latent data structure, we first conducted PCA of the MAS5 normalized expression levels of all the 22,283 experiment-wide gene probes (Fig. 7, Additional file 3: Table S1). PC1 was the unique major PC, accounting for 98.24% of the total variation (Fig. 7a). PC2 merely accounted for 0.32% of total variation. Neither PC1 nor PC2 displayed mean heterogeneity or variance heterogeneity between the smokers and nonsmokers (Fig. 7b). PC4 displayed strikingly significant mean heterogeneity (*p*_*WT*_ = 1.91 × 10^− 15^), even if it only accounted for 0.13% of the total variation. PC6 displayed very significant variance heterogeneity (*p*_*LF*_ = 3.2 × 10^− 4^) even if it accounted for 0.07% of the total variation only. PC4 and PC6 distinguished the smokers and the nonsmokers (Fig. 7c). Additional file 3: Table S1 listed the first 2 and all the global PCs with significant mean and/or variance heterogeneities. These significant global PCs did not distinguish informative heterogeneities and impediment heterogeneities. They were so significant in that they would account for portions of informative mean and variance heterogeneities of DE genes in addition to background heterogeneities. As shown in Fig. 8, naively adjusting for the significant global PCs of all gene probes would result in severe power loss (genomic deflation).

**Fig. 9:**
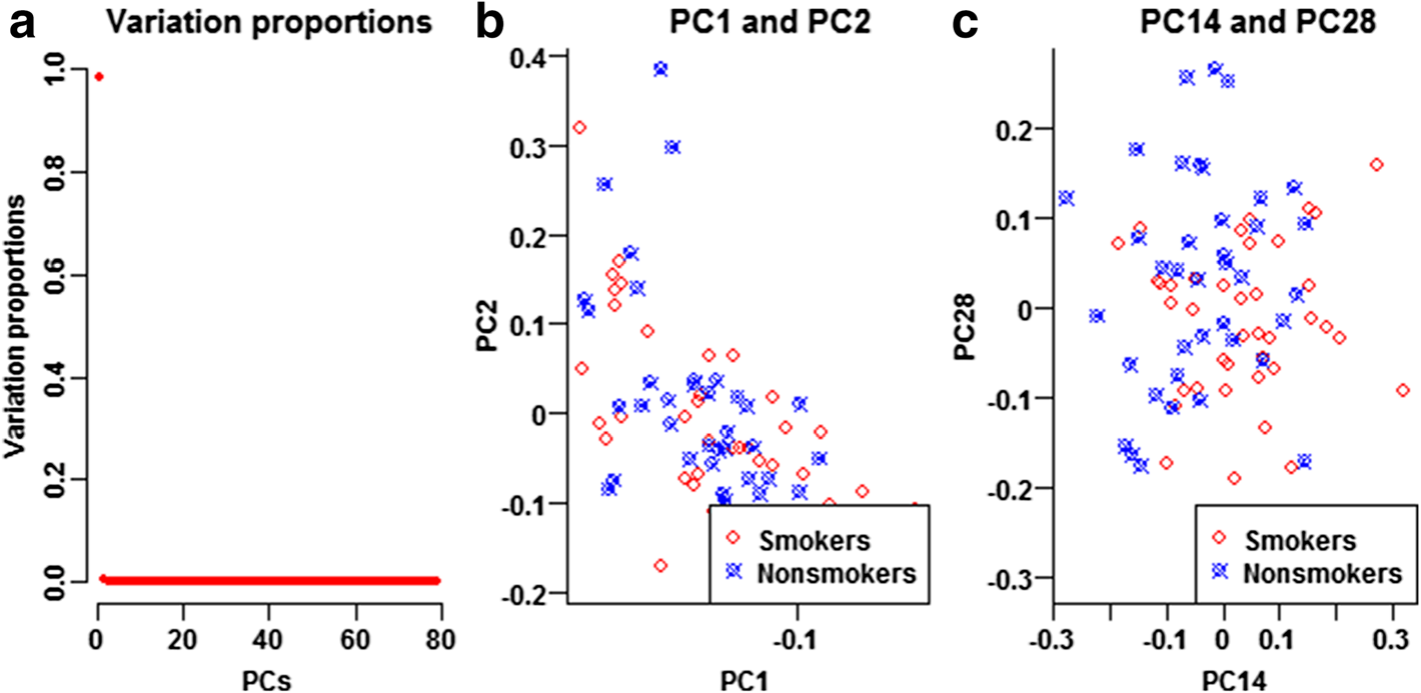
Background data structure of the expression levels of robust gene probes. From the MAS5 normalized data, we selected 13,415 robust gene probes and conducted background PCA. PC1 alone accounted for 98.35% of the total variation and was the unique major PC. PC2 merely accounted for 0.37% of total variation. Neither PC1 nor PC2 displayed mean heterogeneity or variance heterogeneity. PC14 displayed significant mean heterogeneity (*p*_*WT*_ = 0.0036), even if it only accounted for 0.03% of the total variation. PC28 displayed significant variance heterogeneity (*p*_*LF*_ = 0.0069) even if it accounted for 0.01% of the total variation only. PC14 and PC28 displayed clear stratification of the smokers and the nonsmokers. **a** Variation proportions, **b** PC1 and PC2, **c** PC14 and PC28

**Fig. 10:**
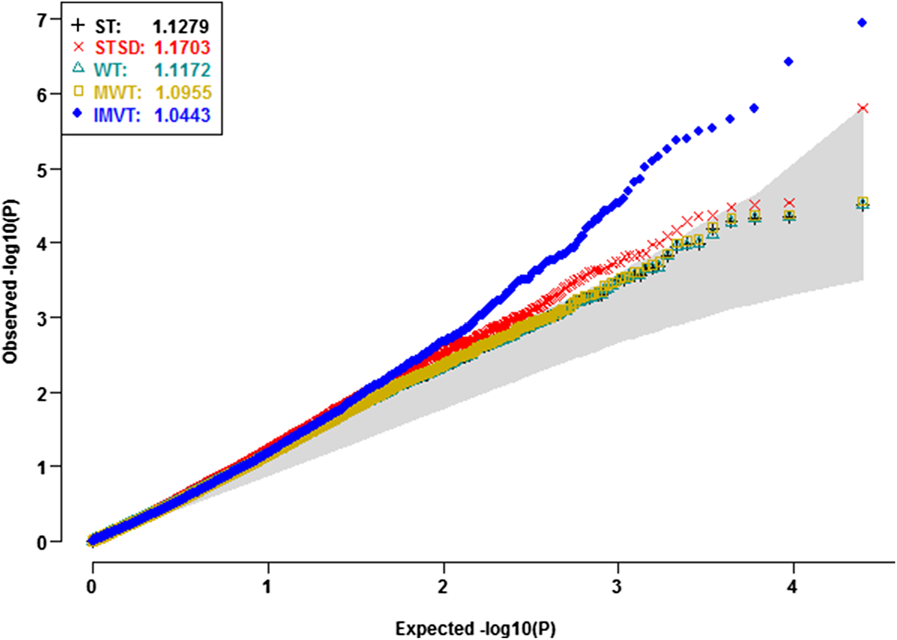
*Q-Q* plots of the five competitors after adjusting for background data structure and covariates. Among all the 79 background PCs, PC14, PC25, PC12, PC16, and PC18 displayed significant mean heterogeneity (*p*_*WT*_ ranged from 0.0036 to 0.0149). PC28, PC29 and PC30 displayed variance heterogeneity (*p*_*LF*_ ranged from 0.0069 to 0.0208). After adjusting for these significant background PCs, age and menopausal status, the *Q-Q* plots of all the five tests climbed above the diagonal. Especially, the Q-Q plot of the IMVT climbed above the upper limit of the 95% concentration band. All the tests displayed reasonable inflation factors. The mild inflation could be due to weak differentials or residual correlations between DE genes. Adjusting for significant background PCs was necessary to prevent false positives and false negatives

To prevent false positives and false negatives, we selected 13,415 ‘robust’ gene probes to capture the background data structure. The spirit here is similar to the use of control genes to account for unwanted variation [17]. None of the robust gene probes displayed mean heterogeneity or variance heterogeneity, before and after calibrating the significant background PCs, age and menopausal status. We conducted PCA of the MAS5 normalized data of the ‘robust’ gene probes (Fig. 9, Additional file 3: Table S2). PC1 alone accounted for 98.35% of the total variation and was the unique major PC. PC2 merely accounted for 0.37% of total variation (Fig. 9a). Neither PC1 nor PC2 displayed mean heterogeneity or variance heterogeneity (Fig. 9b). PC14 displayed the most significant mean heterogeneity (*p*_*WT*_ = 0.0036), even if it only accounted for 0.03% of the total variation. PC28 displayed the most significant variance heterogeneity (*p*_*LF*_ = 0.0069) even if it only accounted for 0.01% of the total variation. PC14 and PC28 displayed clear stratification of the smokers and the nonsmokers (Fig. 9c). In addition, Additional file 3: Table S2 listed the first 2 and all the background PCs with significant mean and/or variance heterogeneities. After adjusting for these significant background PCs, age and menopausal status, the *Q-Q* plots of all the five tests climbed above the diagonal (Fig. 10). Especially, the *Q-Q* plot of the IMVT climbed above the upper limit of the 95% concentration band. All the five tests displayed reasonable inflation factors. The mild inflation might be due to weak differentials or residual correlations between DE genes.

**Fig. 11:**
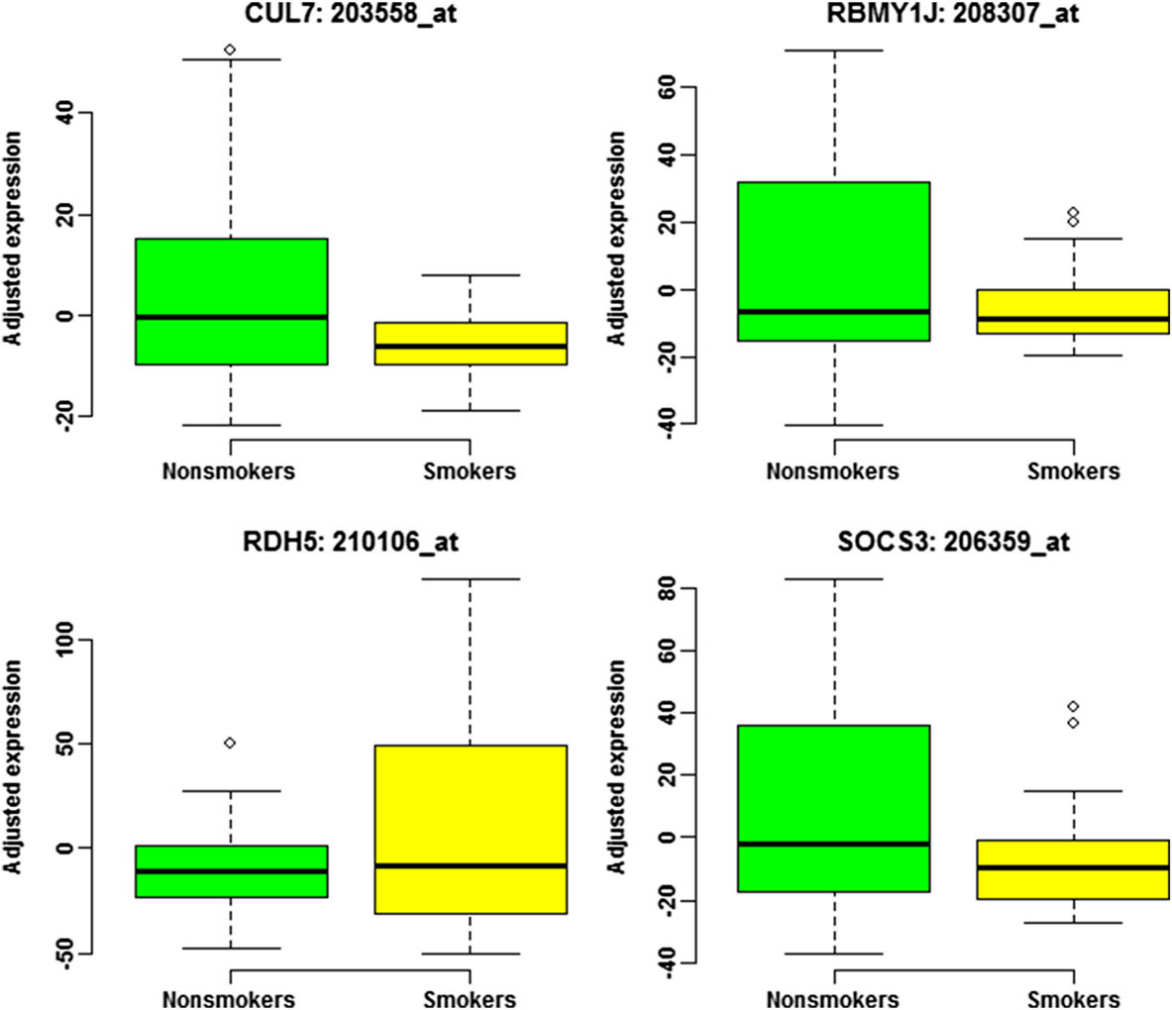
Boxplots of four experiment-wide significant gene probes. After calibrating the background data structure, no gene probes appeared experiment-wide significant mean heterogeneity. All of these four genes displayed certain significance of mean heterogeneity and displayed nearly experiment-wide significant variance heterogeneity. Integrating variance heterogeneity and mean heterogeneity led us to identify these four gene probes to be experiment-wide significant

Applied to the calibrated expressions, our IMVT identified *CUL7, RBMY1J, RDH5* and *SOCS3* to be experiment-wide significant (Table 1), i.e., *p*_*IMVT*_ < 0.05/22283 = 2.24 × 10^− 6^. The STSD only identified *CUL7* as experiment-wide significant gene; while the WT and the MWT failed to identify any experiment-wide significant genes. The experiment-wide minimum *p* value of the WT and the MWT turned to be 2.73 × 10^− 5^, much larger than 2.24 × 10^− 6^. The SMVT failed to identify any gene to be experiment-wide significant. At *DDX3X*, the WT reached the experiment-wide minimum *p*_*WT*_ = 3.10 × 10^− 5^. For SMVT, both *p*_WT_ and *p*_LF_ must be smaller than threshold $1-\sqrt{1-0.05/22283}=1.12\times {10}^{-6}$ to control overall experiment-wide type I error rate at 0.05. Therefore, our analysis of the real data provided solid evidence for the superiority of the IMVT over the SMVT. Without adjusting for the data structure and covariates, Pan et al. [16] did not report any of the four genes although their results were severely inflated. *SOCS3* was reported to be related to tobacco smoking by independent studies [21–24]. Per the database of cancer gene networks (TCNG; http://tcng.hgc.jp/index.html), *CUL7* [25–27], *RBMY1J* [25, 27] and *RDH5* [25–30] were reported to involve in function gene networks related to smoking. All the four experiment-wide significant gene probes displayed both mean and variance heterogeneities (Fig. 11). In addition to the four experiment-wide significant genes, our IMVT identified 16 genes that testified to be involved in functional networks by Pan et al. [16] at nominal level 0.05 (Table 2). For a test gene within a network of functional genes, incorporating its informative variance heterogeneity proved one effective way to exploit extra information as provided by the other function genes in the same network.

**Table 1:** Experiment-wide significant discoveries by the IMVT^a^

AffyID	Gene	IMVT	STSD	MWT	WT
203558_at	*CUL7*	1.12E-07	1.55E-06	0.0034	0.0024
208307_at	*RBMY1J*	3.82E-07	0.0051	0.0422	0.0398
210106_at	*RDH5*	1.56E-06	0.0059	0.0295	0.0302
206359_at	*SOCS3*	2.22E-06	0.0014	0.0081	0.0078

^a^All the probe-specific *p*_*IMVT*_ values reported here are smaller than 0.05/22,283 = 2.2438 × 10^-6^. The STSD identified CUL7 with much weaker evidence while the WT and MWT did not identify any gene probe to be experiment-wide significant

**Table 2:** The overlap of the discoveries of our IMVT and the genes which were testified to be involved in functional networks

AffyID	Gene	Adjusted MAS5*				MAS5**
		IMVT	STSD	MWT	WT	ST
201085_s_at	*SON*	0.0075	0.0021	0.0021	0.0023	2.15E-14
203868_s_at	*VCAM1*	0.0030	0.0004	0.0005	0.0005	2.03E-07
204524_at	*PDPK1*	0.0470	0.0328	0.0337	0.0346	7.12E-11
204600_at	*EPHB3*	0.0178	0.0165	0.0207	0.0213	2.83E-04
205008_s_at	*CIB2*	0.0387	0.0122	0.0117	0.0123	1.25E-06
205099_s_at	*CCR1*	0.0058	0.0104	0.0160	0.0165	6.55E-11
206788_s_at	*CBFB*	0.0003	4.34E-05	4.28E-05	4.71E-05	<1.00E-17
207961_x_at	*MYH11*	0.0001	0.0139	0.0370	0.0383	8.11E-06
208164_s_at	*IL9R*	0.0311	0.0074	0.0072	0.0077	4.05E-05
209876_at	*GIT2*	0.0024	0.0040	0.0053	0.0057	1.20E-08
211197_s_at	*ICOSLG*	0.0448	0.0423	0.0479	0.0487	3.28E-05
211699_x_at	*HBA1*	0.0455	0.3238	0.3632	0.3667	2.70E-03
212514_x_at	*DDX3X*	0.0002	3.06E-05	2.73E-05	3.10E-05	2.22E-16
213446_s_at	*IQGAP1*	0.0082	0.0306	0.0400	0.0413	8.37E-10
217557_s_at	*CPM*	0.0347	0.2422	0.2678	0.2701	1.61E-03
219599_at	*EIF4B*	0.0006	0.0005	0.0018	0.0019	5.80E-14

*These raw *p* values of the heterogeneity tests based on the calibrated expression levels after adjusting for age, menopausal status, and the background structure

*These raw *p* values of Student *t* tests in Pan et al. [16] based on the MAS5 normalized data before adjusting for any of age, menopausal status, and the background structure

The false discovery rate (FDR) would be a more appropriate error rate to control than the familywise error rate in microarray studies; and several standard FDR controlling procedures have been widely practiced [31–34]. We did identify more promising gene probes when applying the most widely used FDR controlling procedure to the *p* values generated by our IMVT. For example, controlling FDR at the stringent level 0.05, our IMVT identified 24 out of the experiment-wide 22,283 gene probes. Controlling FDR at the same level, the STSD only identified *CUL7*, while both the WT and the MWT missed all promising gene probes (Additional file 3: Table S3). Controlling FDR at level 0.1, our IMVT claimed 55 gene probes, while all the three mean heterogeneity tests discovered no additional gene probes. These results have well demonstrated noteworthy gains of explicitly exploiting informative variance heterogeneity. Without adjusting for background data structure, Pant et al. claimed 125 gene probes with local FDRs < 0.05. Their published list of promising gene probes displays huge discrepancies to ours. Such discrepancies stemmed from the severe inflation in their *t* tests (Fig. 6). Judiciously calibrating background data structure is thus necessary for accurately prioritizing gene probes.

## Discussion

Integrating informative variance heterogeneity holds tremendous potential to identify novel genes which involve in gene-gene co-expression and interaction networks. Susceptible genes can co-express as indicated by gene-gene correlations [7, 8]. Genes can interact with each other and/or interact with environmental factors. For example, Pan et al. [16] reported 33 gene probes to involve in constructed functional network. Among which, independent studies reported *MYH11, HOXB1, GIT2, VCAM1, CCR1, IQGAP1, PDPK1, HBA1 HBA2, SON,* and *CPM* to involve in networks related to lung cancer and smoking [25–30]. Within a complex network, the distribution change in the expression level of a single susceptible gene cannot determined by its mean heterogeneity completely. Higher-order heterogeneities can provide extra valuable information for the distribution change. This is why the IMVT led to smaller *p* values than did existent mean heterogeneity tests in our data analyses. In conclusion, integrating informative variance heterogeneity proved an effective step to better capture the latent information conveyed by the co-expression and interaction networks of susceptible genes. It represents one efficient way to extract the inherent higher-order information as induced by complex networks of multiple biomarkers.

The IMVT aims to identify genes whose expression distributions are susceptible to the change in condition. It does not distinguish informative variance heterogeneity from mean heterogeneity. Before applying the IMVT, background data structures must be calibrated to prevent false positive discoveries and power loss. Data structure can be a major confounder for differential analyses, as illustrated by our reanalysis of Pan et al.’s gene profiles [16]. The discrepancy between Pan et al.’s and our discoveries showed the severe confounding impact of the global data structure on differential analyses. In a judicious data calibration, the data structure should be computed from random genes to prevent power loss due to over adjustment.

The IMVT and the SMVT as well, inherit the advantages and disadvantages of the Levene test and the WT. The Levene test is a robust non-parametric method. The exact distribution of the Levene statistic is intractable, and thus its *p*-value must be evaluated by its asymptotic distribution. The condition-specific variance estimators in the Welch statistic could not be accurate for small samples. Thus, the current IMVT is suitable for large samples other than small samples. By our simulation studies and the work of Demissie et al. [6], the MWT could outperform the WT, especially for extremely small sample sizes. Novel parametric methods, i.e., the LRT, are needed to mine expression files of low-replicate experiments. However, the test statistic and its exact null distribution of a parametric test statistic depend on the exact distributions of the (transformed/calibrated) gene expression levels. It is intractable to learn the exact distributions of gene expressions from small samples. Model miss-specifications can mess up differential analyses, as showed by the severe inflations in type I error rate of the normality-based LRT under the Laplace settings. The development of effective small-sample tests requires further formal efforts. In addition, appropriate adjustment of background data structures and other hidden confounders are important for the success of effectively integrating informative variance heterogeneity instead of spurious variance heterogeneity.

Lastly, we acknowledge that there is no need to consider variance heterogeneity in case the distribution of the expression measure of a gene can be determined by a single parameter, i.e., its mean. In such a case, the IMVT can be less powerful than the Welch test. However, single-parameter distribution cannot well fit real-world expression levels in general. Due to the high complexity of gene networks, the expression distribution of a gene cannot be solely determined by its mean. Distribution heterogeneity is a much bigger umbrella than mean heterogeneity. The proposed IMVT merely made one step further from traditional mean heterogeneity tests. High-order heterogeneities are quite common and require particular exploitation methods.

## Conclusions

In this paper, we put forth the concept of MVDE gene and mathematically proved the null independence between mean heterogeneity tests and variance heterogeneity tests. From existent mean heterogeneity tests, we made one step further to identify susceptible genes, whose expression distributions alter with the change in experimental condition. We formally justified this conceptual shift from MDE to MVDE. Specifically, we developed the IMVT as a robust, powerful procedure to integrate informative mean and variance heterogeneities. By extensive simulations under normality and non-normality settings and conducted intensive real-world data analysis, the IMVT outperformed some existent mean heterogeneity tests (e.g., the WT, the MWT, the STSD) and some conventional joint heterogeneity tests (e.g., the LRT, the SMVT).

## Additional files

Additional file 1: Two-sample likelihood ratio test. In this file, we derive the formula of the two-sample likelihood ratio test under the joint null hypothesis. (DOCX 28 kb)
Additional file 2: The null independence between tests statistics on mean and variance heterogeneities. This file is composed of three appendixes. Appendix A: The null independence under normality setting. Appendix B: The null independence under generic spherically symmetric setting. Appendix C: Additional empirical results on the null joint distributions of mean and variance test statistics. (DOCX 36 kb)
Additional file 3: Additional tables of real data analyses. This file displays more results derived by re-analyzing the gene expression profiles of peripheral circulating B Lymphocytes. (DOCX 30 kb)

## Abbreviations

BFWT: Fisher’s linear combination of the Brown-Forsythe test and the Welch *t* test
FWT: Fisher’s linear combination of the *F* test and Welch *t* test
IMVT: Integrative mean-variance test combining Welch *t* test and Levene test
LRT: Likelihood ratio test
MDE gene: Mean differentially expressed gene
MVDE gene: Mean-variance differentially expressed gene
MWT: Moderated Welch *t* test
SMVT: Separate mean-variance testing framework using Welch test and Levene test
ST: Student *t* test
STSD: Student *t* test on condition-wise standardized data
WT: Welch *t* test
