# Erratum to: Integrating mean and variance heterogeneities to identify differentially expressed genes

**DOI:** 10.1186/s12859-017-1507-1

**Published:** 2017-02-03

**Authors:** Weiwei Ouyang, Qiang An, Jinying Zhao, Huaizhen Qin

**Affiliations:** 1 Department of Global Biostatistics and Data Science, Tulane University School of Public Health and Tropical Medicine, 1440 Canal Street, Suite 2001, New Orleans, LA 70112 USA; 2 Department of Epidemiology, Tulane University School of Public Health and Tropical Medicine, 1440 Canal Street, New Orleans, LA 70112 USA; 3 Department of Epidemiology, College of Public Health and Health Professions and College of Medicine, University of Florida, 2004 Mowry Rd, Gainesville, FL 32610 USA

## Erratum

Upon publication of this article [1], an error occurred in the PDF presentation of the hat (WT) formula in ‘Concept of MDE genes and mean heterogeneity tests’ of the ‘Methods’ section on page 3. The corrected formula is shown below:

$$ \widehat{WT}=\frac{{\widehat{\mu}}_{i1}\mathit{\hbox{-}}{\widehat{\mu}}_{i2}}{\sqrt{\frac{{\widehat{\sigma}}_{i1}^2}{n_1}+\frac{{\widehat{\sigma}}_{i2}^2}{n_2}}} $$

The above formula has been corrected in the original article.
