# Supplementary material for: Integrating mean and variance heterogeneities to identify differentially expressed genes
Source: BMC Bioinformatics. 2016 Dec 6;17:497. doi: 10.1186/s12859-016-1393-y (PMC5139036; doi:10.1186/s12859-016-1393-y)
Supplement: Additional file 1: — Two-sample likelihood ratio test. In this file, we derive the formula of the two-sample likelihood ratio test under the joint null hypothesis. (DOCX 28 kb) [file 12859_2016_1393_MOESM1_ESM.docx]

**Two-sample likelihood ratio test**

Herein, we derive the formula of the two-sample likelihood ratio test under the joint null hypothesis. For the $i^{th}$ gene, let $\boldsymbol{G}_{\boldsymbol{i}\boldsymbol{1}}=\left( G_{i11},G_{i21},\ldots,G_{in_{1}1} \right)'$ and $\boldsymbol{G}_{\boldsymbol{i}\boldsymbol{2}}=\left( G_{i12},G_{i22},\ldots,G_{in_{2}2} \right)'$ be expression levels of two independent random samples from normal populations $\mathcal{N}\left( \mu_{i1},\sigma_{i1}^{2} \right)$ and $\mathcal{N}\left( \mu_{i2},\sigma_{i2}^{2} \right)$, respectively. The full likelihood function is given by

$$L\left( \mu_{i1},\mu_{i2},\sigma_{i1}^{2},\sigma_{i2}^{2} \right)$$

$$=\left( \frac{1}{2\pi\sigma_{i1}^{2}} \right)^{\frac{n_{1}}{2}}\left( \frac{1}{2\pi\sigma_{i2}^{2}} \right)^{\frac{n_{2}}{2}}\exp\left( -\frac{1}{2}\left[ \sum_{j=1}^{n_{1}} \left( \frac{G_{ij1}-\mu_{i1}}{\sigma_{i1}} \right)^{2}+\sum_{j=1}^{n_{2}} \left( \frac{G_{ij2}-\mu_{i2}}{\sigma_{i2}} \right)^{2} \right] \right).$$

$$(C1)$$

Under the joint null hypothesis,$\mu_{i1}=\mu_{i2}=\mu_{i}$ and $\sigma_{i1}^{2}=\sigma_{i2}^{2}=\sigma_{i}^{2}$, the reduced likelihood (joint function) can be rewritten as

$$L\left( \mu_{i1},\mu_{i2},\sigma_{i1}^{2},\sigma_{i2}^{2} \right)=L\left( \mu_{i},\mu_{i},\sigma_{i}^{2},\sigma_{i}^{2} \right)$$

$$=\left( \frac{1}{2\pi\sigma_{i}^{2}} \right)^{\frac{n_{1}+n_{2}}{2}}\exp\left( -\frac{1}{2\sigma_{i}^{2}}\left[ \sum_{j=1}^{n_{1}} \left( G_{ij1}-\mu_{i} \right)^{2}+\sum_{j=1}^{n_{2}} \left( G_{ij2}-\mu_{i} \right)^{2} \right] \right). (C2)$$

Solving the system of equations of $\frac{\partial lnL\left( \mu_{i},\mu_{i},\sigma_{i}^{2},\sigma_{i}^{2} \right)}{\partial\mu_{i}}=0$ and $\frac{\partial lnL\left( \mu_{i},\mu_{i},\sigma_{i}^{2},\sigma_{i}^{2} \right)}{\partial\sigma_{i}^{2}}=0$, we derive the maximum likelihood estimators

$$\hat{\mu}_{i}=\frac{1}{n_{1}+n_{2}}\left( \sum_{j=1}^{n_{1}} G_{ij1}+\sum_{j=1}^{n_{2}} G_{ij2} \right)$$

and

$$\hat{\sigma}_{i}^{2}=\frac{1}{n_{1}+n_{2}}\left( \sum_{j=1}^{n_{1}} \left( G_{ij1}-\hat{\mu}_{i} \right)^{2}+\sum_{j=1}^{n_{2}} \left( G_{ij2}-\hat{\mu}_{i} \right)^{2} \right).$$

The maximum of the reduced likelihood $L\left( \mu_{i1},\mu_{i2},\sigma_{i1}^{2},\sigma_{i2}^{2} \right)$ under the joint null hypothesis is

$$\max_{\mu_{i},\sigma_{i}^{2}} L\left( \mu_{i},\mu_{i},\sigma_{i}^{2},\sigma_{i}^{2} \right)=L\left( \hat{\mu}_{i},\hat{\mu}_{i},\hat{\sigma}_{i}^{2},\hat{\sigma}_{i}^{2} \right)$$

$$=\left( \frac{1}{2\pi\hat{\sigma}_{i}^{2}} \right)^{\frac{n_{1}+n_{2}}{2}}\exp\left( -\frac{1}{2\hat{\sigma}_{i}^{2}}\left[ \sum_{j=1}^{n_{1}} \left( G_{ij1}-\hat{\mu}_{i} \right)^{2}+\sum_{j=1}^{n_{2}} \left( G_{ij2}-\hat{\mu}_{i} \right)^{2} \right] \right)$$

$$=\left( \frac{1}{2\pi\hat{\sigma}_{i}^{2}} \right)^{\frac{n_{1}+n_{2}}{2}}\exp\left( -\frac{n_{1}+n_{2}}{2} \right). (C3)$$

Using the full likelihood of $\left( \mu_{i1},\mu_{i2},\sigma_{i1}^{2},\sigma_{i2}^{2} \right)$, we obtain the following system of equations:

$$\left\{ \begin{aligned} \frac{\partial lnL\left( \mu_{i1},\mu_{i2},\sigma_{i1}^{2},\sigma_{i2}^{2} \right)}{\partial\mu_{i1}}=0, \\ \frac{\partial lnL\left( \mu_{i1},\mu_{i2},\sigma_{i1}^{2},\sigma_{i2}^{2} \right)}{\partial\mu_{i2}}=0, \\ \frac{\partial lnL\left( \mu_{i1},\mu_{i2},\sigma_{i1}^{2},\sigma_{i2}^{2} \right)}{\partial\sigma_{i1}^{2}}=0, \\ \frac{\partial lnL\left( \mu_{i1},\mu_{i2},\sigma_{i1}^{2},\sigma_{i2}^{2} \right)}{\partial\sigma_{i2}^{2}}=0. \end{aligned} \right.$$

Solving the system, we derive

$$\hat{\mu}_{i1}=\frac{1}{n_{1}}\sum_{j=1}^{n_{1}} G_{ij1},$$

$$\hat{\mu}_{i2}=\frac{1}{n_{2}}\sum_{j=1}^{n_{2}} G_{ij2},$$

$$\hat{\sigma}_{i1}^{2}=\frac{1}{n_{1}}\sum_{j=1}^{n_{1}} \left( G_{ij1}-\hat{\mu}_{i1} \right)^{2},$$

and

$$\hat{\sigma}_{i2}^{2}=\frac{1}{n_{2}}\sum_{j=1}^{n_{2}} \left( G_{ij2}-\hat{\mu}_{i2} \right)^{2}.$$

Then maximum of $L\left( \mu_{i1},\mu_{i2},\sigma_{i1}^{2},\sigma_{i2}^{2} \right)$ over the full parameter space can be derived as below:

$$\max_{\mu_{i1},\mu_{i2},\sigma_{i1}^{2},\sigma_{i2}^{2}} L\left( \mu_{i1},\mu_{i2},\sigma_{i1}^{2},\sigma_{i2}^{2} \right)=L\left( \hat{\mu}_{i1},\hat{\mu}_{i2},\hat{\sigma}_{i1}^{2},\hat{\sigma}_{i2}^{2} \right)$$

$$=\left( \frac{1}{2\pi\hat{\sigma}_{i1}^{2}} \right)^{\frac{n_{1}}{2}}\left( \frac{1}{2\pi\hat{\sigma}_{i2}^{2}} \right)^{\frac{n_{2}}{2}}\exp\left( -\frac{1}{2}\left[ \sum_{j=1}^{n_{1}} \left( \frac{G_{ij1}-\hat{\mu}_{i1}}{\hat{\sigma}_{i1}} \right)^{2}+\sum_{j=1}^{n_{2}} \left( \frac{G_{ij2}-\hat{\mu}_{i2}}{\hat{\sigma}_{i2}} \right)^{2} \right] \right)$$

$$=\left( \frac{1}{2\pi} \right)^{\frac{n_{1}+n_{2}}{2}}\exp\left( -\frac{n_{1}+n_{2}}{2} \right)\left( \frac{1}{\hat{\sigma}_{i1}^{2}} \right)^{\frac{n_{1}}{2}}\left( \frac{1}{\hat{\sigma}_{i2}^{2}} \right)^{\frac{n_{2}}{2}}. (C4)$$

From eqs. (C3) and (C4), we derive the likelihood ratio

$$LR=\frac{\max_{\mu_{i},\sigma_{i}^{2}} L\left( \mu_{i},\mu_{i},\sigma_{i}^{2},\sigma_{i}^{2} \right)}{\max_{\mu_{i1},\mu_{i2},\sigma_{i1}^{2},\sigma_{i2}^{2}} L\left( \mu_{i1},\mu_{i2},\sigma_{i1}^{2},\sigma_{i2}^{2} \right)}=\frac{\left( \hat{\sigma}_{i1}^{2} \right)^{\frac{n_{1}}{2}}\left( \hat{\sigma}_{i2}^{2} \right)^{\frac{n_{2}}{2}}}{\left( \hat{\sigma}^{2} \right)^{\frac{n_{1}+n_{2}}{2}}}$$

$$=\frac{\left( \frac{1}{n_{1}}\sum_{j=1}^{n_{1}} \left( G_{ij1}-\hat{\mu}_{i1} \right)^{2} \right)^{\frac{n_{1}}{2}}\left( \frac{1}{n_{2}}\sum_{j=1}^{n_{2}} \left( G_{ij2}-\hat{\mu}_{i2} \right)^{2} \right)^{\frac{n_{2}}{2}}}{\left( \frac{1}{n_{1}+n_{2}}\left( \sum_{j=1}^{n_{1}} \left( G_{ij1}-\hat{\mu} \right)^{2}+\sum_{j=1}^{n_{2}} \left( G_{ij2}-\hat{\mu} \right)^{2} \right) \right)^{\frac{n_{1}+n_{2}}{2}}}.$$

For large samples, the statistic $-2ln(LR)$ of likelihood ratio test follows asymptotically chi-square distribution with $\mathrm{df}=2$ under $H_{03}$. The finite-sample performance of the LRT depends on the sample size, and $\chi_{2}^{2}$ distribution may not well approximate the exact distribution of $-2ln(LR)$ for a small sample, which is intractable even under normality setting.
