# Supplementary material for: Integrating mean and variance heterogeneities to identify differentially expressed genes
Source: BMC Bioinformatics. 2016 Dec 6;17:497. doi: 10.1186/s12859-016-1393-y (PMC5139036; doi:10.1186/s12859-016-1393-y)
Supplement: Additional file 2: — The null independence between tests statistics on mean and variance heterogeneities. This file is composed of three appendixes. Appendix A: The null independence under normality setting. Appendix B: The null independence under generic spherically symmetric setting. Appendix C: Additional empirical results on the null joint distributions of mean and variance test statistics. (DOCX 36 kb) [file 12859_2016_1393_MOESM2_ESM.docx]

**Proof and demonstration of theoretical propositions**

The null independence between the test statistics on mean and variance heterogeneities is crucial to warrant the type I error rate control. Herein, we mathematically prove the null independence under normality setting (**Appendix A**) and generic spherically symmetric setting (**Appendix B**). In addition, we empirically demonstrate the joint distributions of the test statistics on mean and variance heterogeneities in **Appendix C**.

**Appendix A: The null independence under normality setting**

At gene $i$, let the two samples of sizes $(n_{1},n_{2})$ follow an identical normal distribution, $N(\mu, \sigma^{2})$. Namely, $H_{03}$ ($\sigma_{i1}^{2}=\sigma_{i2}^{2}=\sigma^{2}$ and $\mu_{i1}=\mu_{i2}=\mu$) is true. Then,

$$Q_{1}≝\frac{\left( n_{1}-1 \right)\hat{\sigma}_{i1}^{2}}{\sigma^{2}}\sim\chi_{n_{1}-1}^{2}, (A1)$$

$$Q_{2}≝\frac{\left( n_{1}-1 \right)\hat{\sigma}_{i2}^{2}}{\sigma^{2}}\sim\chi_{n_{2}-1}^{2}, (A2)$$

$$Z≝\frac{\hat{\mu}_{i1}-\hat{\mu}_{i2}}{\sigma\sqrt{\frac{1}{n_{1}}+\frac{1}{n_{2}}}}\sim N\left( 0,1 \right), (A3)$$

and $Q_{1}, Q_{2}$ and $Z$ are independently distributed. The classical two-sample *F* statistic can be rewritten as

$$\hat{F}=\frac{\hat{\sigma}_{i1}^{2}}{\hat{\sigma}_{i2}^{2}}=\frac{Q_{1}/(n_{1}-1)}{Q_{2}/(n_{2}-1)}\sim F_{n_{1}-1,n_{2}-1}, (A4)$$

The classical two-sample Student *t* statistic can be rewritten as

$$\hat{t}=\frac{\left( \frac{1}{n_{1}}+{\frac{1}{n}}_{2} \right)^{-\frac{1}{2}}\left( \hat{\mu}_{i1}-\hat{\mu}_{i2} \right)}{\sqrt{\frac{n_{1}-1}{n_{1}+n_{2}-2}\hat{\sigma}_{i1}^{2}+\frac{n_{2}-1}{n_{1}+n_{2}-2}\hat{\sigma}_{i2}^{2}}}$$

$$=\frac{Z}{\sqrt{{(Q}_{1}+Q_{2})/(n_{1}+n_{2}-2)}}, (A5)$$

and the two-sample Welch *t* statistic can be rewritten as

$$\hat{t}_{w}=\frac{\left( \hat{\mu}_{i1}-\hat{\mu}_{i2} \right)}{\sqrt{\frac{1}{n_{1}}\hat{\sigma}_{i1}^{2}+\frac{1}{n_{2}}\hat{\sigma}_{i2}^{2}}}$$

$$=\frac{Z}{\sqrt{n_{2}Q_{1}/(n_{1}+n_{2})(n_{1}-1)+n_{1}Q_{2}/(n_{1}+n_{2})(n_{2}-1)}}, (A6)$$

Let $\Gamma(\cdot)$ and $\mathrm{Beta}(\cdot,\cdot)$ denote $\Gamma$ and Beta functions, respectively. By their mutual independence, $Q_{1}$, $Q_{2}$ and $Z$ have joint probability density function

$$p_{\left( Q_{1},Q_{2},Z \right)}\left( q_{1},q_{2},z \right)$$

$$=\frac{q_{1}^{\frac{n_{1}-1}{2}-1}q_{2}^{\frac{n_{2}-1}{2}-1}}{2^{\frac{n_{1}+n_{2}}{2}-1}\Gamma\left( \frac{n_{1}-1}{2} \right)\Gamma\left( \frac{n_{2}-1}{2} \right)}\times\frac{1}{\sqrt{2\pi}}\exp\left( -\frac{q_{1}+q_{2}}{2} \right)\exp\left( -\frac{z^{2}}{2} \right)$$

$$=\frac{q_{1}^{\frac{n_{1}-1}{2}-1}q_{2}^{\frac{n_{2}-1}{2}-1}}{\mathrm{Beta}\left( \frac{n_{1}-1}{2},\frac{n_{2}-1}{2} \right)\mathrm{Beta}\left( \frac{1}{2},\frac{n_{1}+n_{2}}{2}-1 \right)}\frac{\exp\left( -\frac{q_{1}+q_{2}}{2} \right)\exp\left( -\frac{z^{2}}{2} \right)}{2^{\frac{n_{1}+n_{2}-1}{2}}\Gamma\left( \frac{n_{1}+n_{2}-1}{2} \right)}. (A7)$$

By the density formula of a multivariate transformation, the joint probability density function of $\left( \hat{t},\hat{F}, Z \right)$ is given by

$$p_{\left( \hat{t}, \hat{F},Z \right)}\left( t,f,z \right)=p_{\left( Q_{1},Q_{2},Z \right)}\left( q_{1},q_{2},z \right)\times\left| J \right|, (A8)$$

where

$$\left\{ \begin{matrix} q_{1}=\frac{(n_{1}+n_{2}-2)z^{2}}{t^{2}}\frac{(n_{1}-1)f}{\left( n_{1}-1 \right)f+n_{2}-1}, \left( A9 \right) \\ q_{2}=\frac{(n_{1}+n_{2}-2)z^{2}}{t^{2}}\frac{n_{2}-1}{(n_{1}-1)f+n_{2}-1}, (A10) \\ z=z, (A11) \end{matrix} \right.$$

and

$$\left| J \right|=\left\| \frac{\partial\left( q_{1},q_{2},z \right)^{'}}{\partial\left( t,f,z \right)} \right\|=\frac{2(n_{1}-1)(n_{2}-1)}{\left( (n_{1}-1)f+(n_{2}-1) \right)^{2}}\frac{{(n_{1}+n_{2}-2)}^{2}z^{4}}{|{t|}^{5}} (A12)$$

is the absolute Jacobian determinant of the multivariate transformation ( A9, A10 and A11). The support of the joint density of $\hat{t}$, $\hat{F}$ and $Z$ are defined into two sets $\left\{ \left( t,f,z \right) | t>0, f>0, z>0 \right\}$ and $\left\{ \left( t,f,z \right) | t<0, f>0, z<0 \right\}$. Substituting (A9) through (A11) into (A8), we obtain the joint density of $\left( \hat{t},\hat{F} \right)$ by integrating variable $Z$

$$p_{\left( \hat{t}, \hat{F} \right)}\left( t,f \right)=\int_{-\infty}^{\infty} p_{\left( \hat{t}, \hat{F},Z \right)}\left( t,f,z \right)\mathrm{dz}$$

$$=\int_{-\infty}^{\infty} p_{\left( Q_{1},Q_{2},Z \right)}\left( q_{1},q_{2},z \right)\times\left| J \right|\mathrm{dz}$$

$$=\frac{\frac{1}{f}\left( \frac{\left( \left( n_{1}-1 \right)f \right)^{\left( n_{1}-1 \right)}\left( n_{2}-1 \right)^{n_{2}-1}}{\left( \left( n_{1}-1 \right)f+n_{2}-1 \right)^{2}} \right)^{\frac{1}{2}}}{\mathrm{Beta}\left( \frac{1}{2},\frac{n_{1}+n_{2}}{2}-1 \right)\mathrm{Beta}\left( \frac{n_{1}-1}{2},\frac{n_{2}-1}{2} \right)}$$

$$\times\int_{0}^{\infty} \frac{\left( \frac{\left( n_{1}+n_{2}-2 \right)z^{2}}{t^{2}} \right)^{\frac{n_{1}+n_{2}}{2}-1}\exp\left\{ -\frac{z^{2}}{2}\left( 1+\frac{n_{1}+n_{2}-2}{t^{2}} \right) \right\}}{2^{\frac{n_{1}+n_{2}-3}{2}}\Gamma\left( \frac{n_{1}+n_{2}-1}{2} \right)\left| t \right|}dz$$

$$=p_{\hat{F}}\left( f \right)\times p_{\hat{t}}\left( t \right), \left( A13 \right)$$

where

$$p_{\hat{F}}\left( f \right)=\frac{\frac{1}{f}\left( \frac{\left( (n_{1}-1)f \right)^{(n_{1}-1)}{(n_{2}-1)}^{(n_{2}-1)}}{\left( \left( n_{1}-1 \right)f+n_{2}-1 \right)^{2}} \right)^{\frac{1}{2}}}{\mathrm{Beta}\left( \frac{n_{1}-1}{2},\frac{n_{2}-1}{2} \right)} (A14)$$

is the probability density function of the $F$ statistic, and

$$p_{\hat{t}}\left( t \right)=\frac{\left( 1+\frac{t^{2}}{n_{1}+n_{2}-2} \right)^{-\frac{n_{1}+n_{2}-1}{2}}}{\sqrt{n_{1}+n_{2}-2}\mathrm{Beta}\left( \frac{1}{2},\frac{n_{1}+n_{2}}{2}-1 \right)} (A15)$$

is the probability density function of the Student $t$ statistic. In summary, if $H_{03}$ holds, then $\hat{F}$ and $\hat{t}$ are independently distributed.

Under the normality setting, the null independence of Welch *t* statistic to F statistics can be similarly proved. Specifically, we only need to consider transformation system

$$\left\{ \begin{matrix} q_{1}=\frac{(n_{1}+n_{2})z^{2}}{t_{w}^{2}}\frac{(n_{1}-1)f}{n_{2}f+n_{1}}, \left( A16 \right) \\ q_{2}=\frac{(n_{1}+n_{2})z^{2}}{t_{w}^{2}}\frac{n_{2}-1}{n_{2}f+n_{1}}, (A17) \\ z=z. (A18) \end{matrix} \right.$$

Substituting (A16) through (A18) into (A8) and repeating the other steps can prove the null independence between F statistic and Welch t statistic.

**Appendix B: The null independence between tests statistics on mean and variance heterogeneities under generic spherically symmetric setting**

Mean heterogeneity tests in two-sample comparisons can be equivalent to a simple linear regression model:

$G_{ij}=\beta_{0i}+\beta_{i}K_{j}+e_{ij}, (B1)$

where $G_{ij}$ is the expression level of the $i^{th}$ gene of the $j^{th}$ subject, $K_{j}=1$ if the $j^{th}$ subject belongs to Group 1, and $K_{j}=0$ if otherwise,$\beta_{0i}$ is intercept and $\beta_{i}$ is the effect of group on gene expression levels, and $e_{ij}$ is random error. According to ordinary least squares (OLS) method, we obtain $\hat{\beta}_{i}=\hat{\mu}_{i1}-\hat{\mu}_{i2}$ and $\hat{\beta}_{0i}=\hat{\mu}_{i2}$. The standard error of $\hat{\beta}_{i}$ is

$$\mathrm{SE}_{\hat{\beta}_{i}}=\sqrt{\left( \frac{1}{n_{1}+n_{2}-2} \right)\left( \frac{\sum_{j=1}^{n_{1}+n_{2}} \left( G_{ij}-\hat{G}_{ij} \right)^{2}}{\sum_{j=1}^{n_{1}+n_{2}} \left( K_{j}-\bar{K}_{j} \right)^{2}} \right)}$$

$$=\sqrt{\left( \frac{1}{n_{1}+n_{2}-2} \right)\left( \frac{\sum_{j=1}^{n_{1}} \left( G_{ij1}-\hat{\mu}_{i1} \right)^{2}+\sum_{j=1}^{n_{1}} \left( G_{ij1}-\hat{\mu}_{i1} \right)^{2}}{\sum_{j=1}^{n_{1}+n_{2}} \left( K_{j}-\bar{K}_{j} \right)^{2}} \right)}$$

$$=\sqrt{\left( \frac{1}{n_{1}+n_{2}-2} \right)\left( \frac{\left( n_{1}-1 \right)\hat{\sigma}_{i1}^{2}+\left( n_{2}-1 \right)\hat{\sigma}_{i2}^{2}}{\frac{n_{1}n_{2}}{n_{1}+n_{2}}} \right)} , (B2)$$

where $\hat{G}_{ij}=\hat{\beta}_{0i}+\hat{\beta}_{i}K_{j}$. The statistic to test $\beta_{i}=0$ in $(B1)$ can be written as

$$t_{regression}=\frac{\hat{\beta}_{i}}{\mathrm{SE}_{\hat{\beta}_{i}}}$$

$$=\frac{\left( \frac{1}{n_{1}}+{\frac{1}{n}}_{2} \right)^{-\frac{1}{2}}\left( \hat{\mu}_{i1}-\hat{\mu}_{i2} \right)}{\sqrt{\frac{n_{1}-1}{n_{1}+n_{2}-2}\hat{\sigma}_{i1}^{2}+\frac{n_{2}-1}{n_{1}+n_{2}-2}\hat{\sigma}_{i2}^{2}}}. (B3)$$

Thus, it is mathematically the Student *t* statistic in two-sample group comparisons. Under spherically symmetric distribution conditions, the density of gene expression levels is

$$L_{G}=\prod_{j=1}^{(n_{1}+n_{2})} \frac{1}{\sigma}g\left( \frac{\left( G_{ij}-E\left( G_{ij} \right) \right)^{2}}{\sigma^{2}} \right)$$

$$=\frac{1}{\sigma^{n_{1}+n_{2}}}g\left( \sum_{j=1}^{\left( n_{1}+n_{2} \right)} \frac{\left( G_{ij}-E\left( G_{ij} \right) \right)^{2}}{\sigma^{2}} \right), (B4)$$

where $g(.)$ is a given monotone function called the generating function with respect to the Lebesgue measure in $\mathbb{R}$, $E\left( G_{ij} \right)=\beta_{0i}+\beta_{i}K_{j}$ is the conditional expectation given $K_{j}$. Similar to the theorem for exponential family in Lehmann’s book [44], the complete sufficient statistic for gene expression distribution is $\boldsymbol{T}=\left( \sum_{j=1}^{n_{1}} G_{ij1}^{2}+\sum_{j=1}^{n_{2}} G_{ij2}^{2},\sum_{j=1}^{n_{1}} G_{ij1}+\sum_{j=1}^{n_{2}} G_{ij2},\sum_{j=1}^{n_{1}} G_{ij1} \right)$. Note that the *t* statistic of mean heterogeneity test is a function of $\boldsymbol{T}$. In addition, the *LF* statistic of Levene’s test approximately follows *F* distribution with 1 and $(n_{1}+n_{2}-2)$ degree of freedoms. And this F distribution does not depend on parameters $\beta_{0i},\beta_{i}, \sigma^{2}$ in (*B*1). Therefore, according to Basu’s theorem [1], the *LF* and the Student t statistics are independently distributed ($\hat{LF}⫫\hat{t}$). Within the family of spherically symmetric distributions, mean and mode is the same and thus the BF statistic is also independent of Student t statistic ( $\hat{BF}⫫\hat{t}$). $\hat{LF}⫫\hat{t}_{w}$ and $\hat{BF}⫫\hat{t}_{w}$ can be similarly proved. Since spherically symmetric distribution family is a very broad distribution family that include spherical exponential family, Student distribution, Laplace distribution, exponential power distribution and many other distributions, the Student and Welch t-statistics are independent of the Levene and Brown-Forsythe statistics under normality settings by letting random error $e$ follow normal distribution in (*B*1).

**Additional Reference**

1. Casella G, Berger RL: **Statistical inference**, vol. 2: Duxbury Pacific Grove, CA; 2002.

**Appendix C: Additional empirical results on the null joint distributions of mean and variance test statistics**

**Figure S1.1: Null joint distributions of mean and variance test statistics under 5 vs. 5 normality setting.** Each panels displays 100000 pairs of the specified test statistics, which were computed from 100000 replicates of two-group samples of sizes ($n_{1}=n_{2}=5$) from the standard normal distribution. Panel (**a**) shows the null independence between Welch *t* statistic and Levene statistic. Panel (**b**) shows the null independence between Welch *t-*statistic and *F*-statistic. Panel (**c**) shows the equivalence between Welch *t* statistic and Student t statistic. Panel (**d**) shows the high correlation between Levene test statistic and Brown-Forsythe statistic.

**Figure S1.2: Null joint distributions of mean and variance test statistics under 10 vs. 10 normality setting.** Each panels displays 100000 pairs of the specified test statistics, which were computed from 100000 replicates of two-group samples of sizes ($n_{1}=n_{2}=10$) from the standard normal distribution. Panel (**a**) shows the null independence between Welch *t* statistic and Levene statistic. Panel (**b**) shows the null independence between Welch *t-*statistic and *F*-statistic. Panel (**c**) shows the equivalence between Welch *t* statistic and Student t statistic. Panel (**d**) shows the high correlation between Levene test statistic and Brown-Forsythe statistic.

**Figure S1.3: Null joint distributions of mean and variance test statistics under 20 vs. 20 normality setting.** Each panels displays 100000 pairs of the specified test statistics, which were computed from 100000 replicates of two-group samples of sizes ($n_{1}=n_{2}=20$) from the standard normal distribution. Panel (**a**) shows the null independence between Welch *t* statistic and Levene statistic. Panel (**b**) shows the null independence between Welch *t-*statistic and *F*-statistic. Panel (**c**) shows the equivalence between Welch *t* statistic and Student t statistic. Panel (**d**) shows the high correlation between Levene test statistic and Brown-Forsythe statistic.

**Figure S2.1: Null joint distributions of mean and variance test statistics under 5 vs. 5 Laplace setting.** Each panels displays 100000 pairs of the specified test statistics, which were computed from 100000 replicates of two-group samples of sizes ($n_{1}=n_{2}=5$) from the standard Laplace distribution. Panel (**a**) shows the null independence between Welch *t* statistic and Levene statistic. Panel (**b**) shows the null independence between Welch *t-*statistic and *F*-statistic. Panel (**c**) shows the equivalence between Welch *t* statistic and Student t statistic. Panel (**d**) shows the high correlation between Levene test statistic and Brown-Forsythe statistic.

**Figure S2.2: Null joint distributions of mean and variance test statistics under 10 vs. 10 Laplace setting.** Each panels displays 100000 pairs of the specified test statistics, which were computed from 100000 replicates of two-group samples of sizes ($n_{1}=n_{2}=10$) from the standard Laplace distribution. Panel (**a**) shows the null independence between Welch *t* statistic and Levene statistic. Panel (**b**) shows the null independence between Welch *t-*statistic and *F*-statistic. Panel (**c**) shows the equivalence between Welch *t* statistic and Student t statistic. Panel (**d**) shows the high correlation between Levene test statistic and Brown-Forsythe statistic.

**Figure S2.3: Null joint distributions of mean and variance test statistics under 20 vs. 20 Laplace setting.** Each panels displays 100000 pairs of the specified test statistics, which were computed from 100000 replicates of two-group samples of sizes ($n_{1}=n_{2}=20$) from the standard Laplace distribution. Panel (**a**) shows the null independence between Welch *t* statistic and Levene statistic. Panel (**b**) shows the null independence between Welch *t-*statistic and *F*-statistic. Panel (**c**) shows the equivalence between Welch *t* statistic and Student t statistic. Panel (**d**) shows the high correlation between Levene test statistic and Brown-Forsythe statistic.

**Figure S2.4: Null joint distributions of mean and variance test statistics under 40 vs. 40 Laplace setting.** Each panels displays 100000 pairs of the specified test statistics, which were computed from 100000 replicates of two-group samples of sizes ($n_{1}=n_{2}=40$) from the standard Laplace distribution. Panel (**a**) shows the null independence between Welch *t* statistic and Levene statistic. Panel (**b**) shows the null independence between Welch *t-*statistic and *F*-statistic. Panel (**c**) shows the equivalence between Welch *t* statistic and Student t statistic. Panel (**d**) shows the high correlation between Levene test statistic and Brown-Forsythe statistic.
