# Supplementary material for: Integrating mean and variance heterogeneities to identify differentially expressed genes
Source: BMC Bioinformatics. 2016 Dec 6;17:497. doi: 10.1186/s12859-016-1393-y (PMC5139036; doi:10.1186/s12859-016-1393-y)
Supplement: Additional file 3: — Additional tables of real data analyses. This file displays more results derived by re-analyzing the gene expression profiles of peripheral circulating B Lymphocytes. (DOCX 30 kb) [file 12859_2016_1393_MOESM3_ESM.docx]

**Additional tables of real data analyses**

In this file, we provide more tables as derived by re-analyzing the gene expression profiles of peripheral circulating B Lymphocytes

| **Table S1. The first 2 and significant PCs of all the experiment-wide gene probes** | | | | | | |
| --- | --- | --- | --- | --- | --- | --- |
| **PC Index** | **Eigen**  **Value** | **Variation Proportion** | ***P* values of** | | | |
|  |  |  | **ST** | **WT** | **MWT** | **Levene** |
| 1 | 1.48E+13 | **0.9824** | 0.5343 | 0.5355 | 0.8459 | 0.2359 |
| 2 | 4.89E+10 | **0.0032** | 0.5786 | 0.5782 | 0.5752 | 0.9243 |
| **4** | 2.01E+10 | 0.0013 | **1.84E-15** | **1.91E-15** | **4.49E-15** | 0.0935 |
| **6** | 1.08E+10 | 0.0007 | 0.1148 | 0.1134 | 0.1102 | **0.0003** |
| **9** | 7.15E+09 | 0.0005 | 0.9725 | 0.9723 | 0.972 | **0.0015** |
| **14** | 4.32E+09 | 0.0003 | 0.5616 | 0.5598 | 0.557 | **0.0114** |
| **28** | 1.88E+09 | 0.0001 | 0.5533 | 0.5519 | 0.549 | **0.0185** |
| **38** | 1.33E+09 | 0.0001 | 0.9091 | 0.9095 | 0.9084 | **0.0419** |
| **49** | 9.81E+08 | 0.0001 | 0.3012 | 0.2993 | 0.2958 | **0.0142** |
| **78** | 4.47E+08 | 2.97E-05 | 0.8199 | 0.8217 | 0.8189 | **0.0129** |

| **Table S2. The first 2 and significant PCs of 13415 experiment-wide robust gene probes** | | | | | | |
| --- | --- | --- | --- | --- | --- | --- |
| **PC Index** | **Eigen**  **Value** | **variation Proportion** | ***P* values of** | | | |
|  |  |  | **ST** | **WT** | **MWT** | **Levene** |
| 1 | 9.06E+12 | **0.9835** | 0.6702 | 0.6712 | 0.8922 | 0.1865 |
| 2 | 3.40E+10 | **0.0037** | 0.6448 | 0.6442 | 0.6419 | 0.6613 |
| **12** | 2.89E+09 | 0.0003 | **0.0064** | **0.0063** | **0.006** | 0.0557 |
| **14** | 2.36E+09 | 0.0003 | **0.0035** | **0.0036** | **0.0033** | 0.538 |
| **16** | 2.03E+09 | 0.0002 | **0.0135** | **0.0134** | **0.0129** | 0.3799 |
| **18** | 1.67E+09 | 0.0002 | **0.0151** | **0.0149** | **0.0144** | 0.1453 |
| **25** | 1.17E+09 | 0.0001 | **0.0059** | **0.0058** | **0.0056** | 0.9441 |
| **28** | 1.01E+09 | 0.0001 | 0.2272 | 0.2253 | 0.222 | **0.0069** |
| **29** | 9.91E+08 | 0.0001 | 0.6090 | 0.6074 | 0.605 | **0.0119** |
| **30** | 9.78E+08 | 0.0001 | 0.9674 | 0.9675 | 0.9672 | **0.0208** |

| **Table S3. Discoveries of the IMVT by controlling FDR below 0.1** | | | | | |
| --- | --- | --- | --- | --- | --- |
| **AffyID** | **Gene** | **Local FDR** | | | |
|  |  | **IMVT** | **STSD** | **MWT** | **WT** |
| 203558_at | *CUL7* | 0.0025 | 0.0344 | 0.4676 | 0.4701 |
| 208307_at | *RBMY1J* | 0.0043 | 0.3490 | 0.6452 | 0.6345 |
| 204384_at | *GOLGA2* | 0.0116 | 0.2158 | 0.4607 | 0.4701 |
| 206359_at | *SOCS3* | 0.0116 | 0.3158 | 0.4766 | 0.4915 |
| 208294_x_at | *CSHL1* | 0.0116 | 0.2155 | 0.4445 | 0.4701 |
| 210106_at | *RDH5* | 0.0116 | 0.3568 | 0.6046 | 0.6039 |
| 214436_at | *FBXL2* | 0.0116 | 0.7192 | 0.8635 | 0.8527 |
| 218922_s_at | *CERS4* | 0.0116 | 0.3158 | 0.4761 | 0.4915 |
| 214886_s_at | *N4BP2L1* | 0.0137 | 0.3231 | 0.7155 | 0.6722 |
| 210492_at | *MFAP3L* | 0.0160 | 0.3306 | 0.6466 | 0.6267 |
| 206162_x_at | *SYT5* | 0.0162 | 0.4133 | 0.6414 | 0.6317 |
| 215840_at | *DNAH2* | 0.0176 | 0.2158 | 0.5222 | 0.5227 |
| 214257_s_at | *SEC22B* | 0.0240 | 0.2158 | 0.4445 | 0.4701 |
| 219829_at | *ITGB1BP2* | 0.0249 | 0.3953 | 0.6717 | 0.6557 |
| 211789_s_at | *MLXIP* | 0.0305 | 0.3158 | 0.6595 | 0.6430 |
| 209461_x_at | *WDR18* | 0.0359 | 0.6251 | 0.7799 | 0.7725 |
| 210974_s_at | *AP3D1* | 0.0373 | 0.2457 | 0.4445 | 0.4701 |
| 214145_s_at | *SPTB* | 0.0373 | 0.3231 | 0.4761 | 0.4915 |
| 210922_at | *BC000772* | 0.0397 | 0.5364 | 0.7180 | 0.7128 |
| 220625_s_at | *ELF5* | 0.0404 | 0.3134 | 0.4676 | 0.4788 |
| 222260_at | *AK026947* | 0.0404 | 0.3306 | 0.5112 | 0.5224 |
| 203532_x_at | *CUL5* | 0.0441 | 0.3810 | 0.6342 | 0.6281 |
| 214138_at | *ZNF79* | 0.0460 | 0.5952 | 0.7812 | 0.7772 |
| 221208_s_at | *MSANTD2* | 0.0460 | 0.1610 | 0.2808 | 0.3123 |
| 203609_s_at | *ALDH5A1* | 0.0514 | 0.3271 | 0.4766 | 0.4915 |
| 222256_s_at | *JMJD7* | 0.0517 | 0.3402 | 0.5112 | 0.5179 |
| 204947_at | *E2F1* | 0.0544 | 0.2158 | 0.4766 | 0.4915 |
| 214803_at | *CDH6* | 0.0643 | 0.3450 | 0.6213 | 0.6184 |
| 221528_s_at | *ELMO2* | 0.0643 | 0.4408 | 0.7337 | 0.7364 |
| 218659_at | *ASXL2* | 0.0710 | 0.3134 | 0.4761 | 0.4915 |
| 209666_s_at | *CHUK* | 0.0783 | 0.3158 | 0.4676 | 0.4788 |
| 203918_at | *PCDH1* | 0.0808 | 0.3262 | 0.5112 | 0.5224 |
| 208524_at | *GPR15* | 0.0808 | 0.1610 | 0.2748 | 0.3123 |
| 209850_s_at | *CDC42EP2* | 0.0816 | 0.3297 | 0.5033 | 0.5175 |
| 204854_at | *LEPREL2* | 0.0848 | 0.3946 | 0.6046 | 0.6031 |
| 206604_at | *OVOL1* | 0.0848 | 0.3490 | 0.5143 | 0.5227 |
| 207961_x_at | *MYH11* | 0.0848 | 0.4337 | 0.6289 | 0.6317 |
| 216975_x_at | *NPAS1* | 0.0848 | 0.3231 | 0.5947 | 0.5859 |
| 222015_at | *CSNK1E* | 0.0848 | 0.2005 | 0.4445 | 0.4566 |
| 200080_s_at | *H3F3AP4* | 0.0886 | 0.1912 | 0.2808 | 0.3123 |
| 205391_x_at | *ANK1* | 0.0886 | 0.3953 | 0.5947 | 0.5921 |
| 209156_s_at | *COL6A2* | 0.0886 | 0.3810 | 0.6897 | 0.6713 |
| 210565_at | *GCGR* | 0.0886 | 0.3231 | 0.5161 | 0.5227 |
| 216006_at | *AF070620* | 0.0886 | 0.3231 | 0.4761 | 0.4915 |
| 216584_at | *216584_at* | 0.0886 | 0.3134 | 0.4873 | 0.4915 |
| 219733_s_at | *SLC27A5* | 0.0886 | 0.3158 | 0.4761 | 0.4915 |
| 205387_s_at | *CGB7* | 0.0905 | 0.3490 | 0.5267 | 0.5314 |
| 222084_s_at | *SBF1* | 0.0924 | 0.3158 | 0.4676 | 0.4788 |
| 206298_at | *ARHGAP22* | 0.0955 | 0.3564 | 0.5847 | 0.5854 |
| 207150_at | *SLC18A3* | 0.0969 | 0.3262 | 0.4761 | 0.4915 |
| 215786_at | *AK022170* | 0.0969 | 0.2158 | 0.4094 | 0.4535 |
| 219729_at | *PRRX2* | 0.0969 | 0.3490 | 0.5847 | 0.5793 |
| 220735_s_at | *SENP7* | 0.0969 | 0.3231 | 0.4761 | 0.4915 |
| 216313_at | *PCDHB17* | 0.0972 | 0.2242 | 0.4980 | 0.4933 |
| 212514_x_at | *DDX3X* | 0.0990 | 0.1610 | 0.2595 | 0.2962 |
